# Single amino acid–based PROTACs trigger degradation of the oncogenic kinase BCR–ABL in chronic myeloid leukemia (CML)

**DOI:** 10.1016/j.jbc.2023.104994

**Published:** 2023-06-29

**Authors:** Jianchao Zhang, Caibing Ma, Yongjun Yu, Chaowei Liu, Lijing Fang, Hai Rao

**Affiliations:** 1 Department of Biochemistry, School of Medicine, Southern University of Science and Technology, Shenzhen, China; 2 Institute of Biomedicine and Biotechnology, Shenzhen Institute of Advanced Technology, Chinese Academy of Sciences, Shenzhen, Guangdong, China; 3 Key University Laboratory of Metabolism and Health of Guangdong, Southern University of Science and Technology, Shenzhen, China

**Keywords:** PROTAC, N-end rule, amino acid, leukemia, protein degradation, drug design, ubiquitin, cancer

## Abstract

Proteolysis-targeting chimera (PROTAC) that specifically targets harmful proteins for destruction by hijacking the ubiquitin–proteasome system is emerging as a potent anticancer strategy. How to efficiently modulate the target degradation remains a challenging issue. In this study, we employ a single amino acid–based PROTAC, which uses the shortest degradation signal sequence as the ligand of the N-end rule E3 ubiquitin ligases to degrade the fusion protein BCR (breakpoint cluster region)–ABL (Abelson proto-oncogene), an oncogenic kinase that drives the progression of chronic myeloid leukemia. We find that the reduction level of BCR–ABL can be easily adjusted by substituting different amino acids. Furthermore, a single PEG linker is found to achieve the best proteolytic effect. Our efforts have resulted in effective degradation of BCR–ABL protein by the N-end rule pathway and efficient growth inhibition of K562 cells expressing BCR–ABL *in vitro* and blunted tumor growth in a K562 xenograft tumor model *in vivo*. The PROTAC presented has unique advantages including lower effective concentration, smaller molecular size, and modular degradation rate. Demonstrating the efficacy of the N-end rule–based PROTACs *in vitro* and *in vivo*, our study further expands the limited degradation pathways currently available for PROTACs *in vivo* and is easily adapted for broader applications in targeted protein degradation.

Chronic myeloid leukemia (CML) is a type of hematological malignancy that is often caused by the BCR (breakpoint cluster region)–ABL (Abelson proto-oncogene), a fusion product of BCR and ABL kinase triggered by chromosome translocation (1, 2). The aberrant BCR–ABL fusion leads to abnormally higher tyrosine kinase activity, partially because of enhanced protein stability, which in turn induces the uncontrolled proliferation of myeloid cells in the bone marrow and triggers leukemia (3, 4). The BCR–ABL tyrosine kinase inhibitors (TKIs) such as imatinib and dasatinib (Dasa) have been demonstrated to be efficient in locking BCR–ABL into an inactive conformation and thereby block its kinase activity (5, 6). These TKIs are Food and Drug Administration–approved drugs effective for CML. However, drug resistance often occurs within a year, limiting its long-term efficacy, a common shortcoming for many targeted therapies (7, 8). A major reason behind the acquired resistance is that BCR–ABL inhibition requires persistent binding of imatinib or Dasa, which provides time for cells to develop alternative route to resume cell growth (5, 9).

Proteolysis-targeting chimera (PROTAC) has emerged as a promising therapeutic strategy to modulate protein stability that would not only inhibit but also rapidly eliminate target protein on contact, likely improving drug efficiency and alleviating drug resistance (10, 11, 12, 13). Bridged by a suitable linker (*e.g.*, PEG) in the middle, PROTACs consist of a moiety for recruiting an E3 ubiquitin (Ub) ligase and a ligand for the target protein, bringing an E3 Ub ligase to a target protein to trigger substrate ubiquitylation and subsequent destruction (10, 11, 12, 13). Although the ligands for target proteins have been extensively developed previously, the degradation motifs responsible for recruiting E3 ligase remain underdeveloped for the application in PROTACs (13, 14, 15). Thus far, the degradation signals have been largely restricted to two E3 Ub ligases, VHL (∼30%) and CRBN (∼60%) (16), which have certain limitations because of the cell- or tissue-specific expression (17). Moreover, the poor cell permeability of PROTACs caused by larger molecular weights impedes their access to intracellular targets (18). An ideal PROTAC is also to maintain the amount of target proteins in the right range as too much or little substrate turnover may be detrimental in some instances; and yet nearly all the PROTACs currently available lack degradation rate modulation. Therefore, despite the recent successful clinical trials, how to improve the applicability of this technique remains a major challenge.

We have developed the PROTACs based on the N-end rule degradation pathway, which ties the stability of a protein to the identity of its N-terminal residue (19, 20, 21, 22). Single amino acids are the first and simplest degradation signal sequences uncovered in 1980s (20, 22). Presented as the first amino acid, a basic (*i.e.*, Arg, Lys, His) or hydrophobic (*i.e.*, Leu, Ile, Phe, Trp, Tyr) residue can be directly recognized by the UBR1 family of E3 Ub ligases, which then induce substrate ubiquitylation and degradation. As we have demonstrated that enhanced stability of BCR–ABL contributes to its oncogenic potential (4), PROTACs present an effective means to keep BCR–ABL in check. We appended four amino acids (*i.e.*, Arg, Lys, Leu, and Phe) separately to Dasa, a BCR–ABL ligand, with PEG as the linker. These PROTACs induce the degradation of BCR–ABL with varying degree of efficiency in the nanomolar range and inhibit the cellular proliferation of K562 with IC_50_ values less than 0.5 nM *in vitro*. The Arg-PEG1-Dasa PROTAC exhibits robust antitumor effects in K562 xenograft mouse model *in vivo*. These findings demonstrate the efficacy of single amino acid–based PROTACs *in vitro* and *in vivo*, highlighting its advantage and potential in the development of protein knockdown–based therapy.

## Results

### Design and synthesis of BCR–ABL–targeting PROTACs

**Figure 1:**
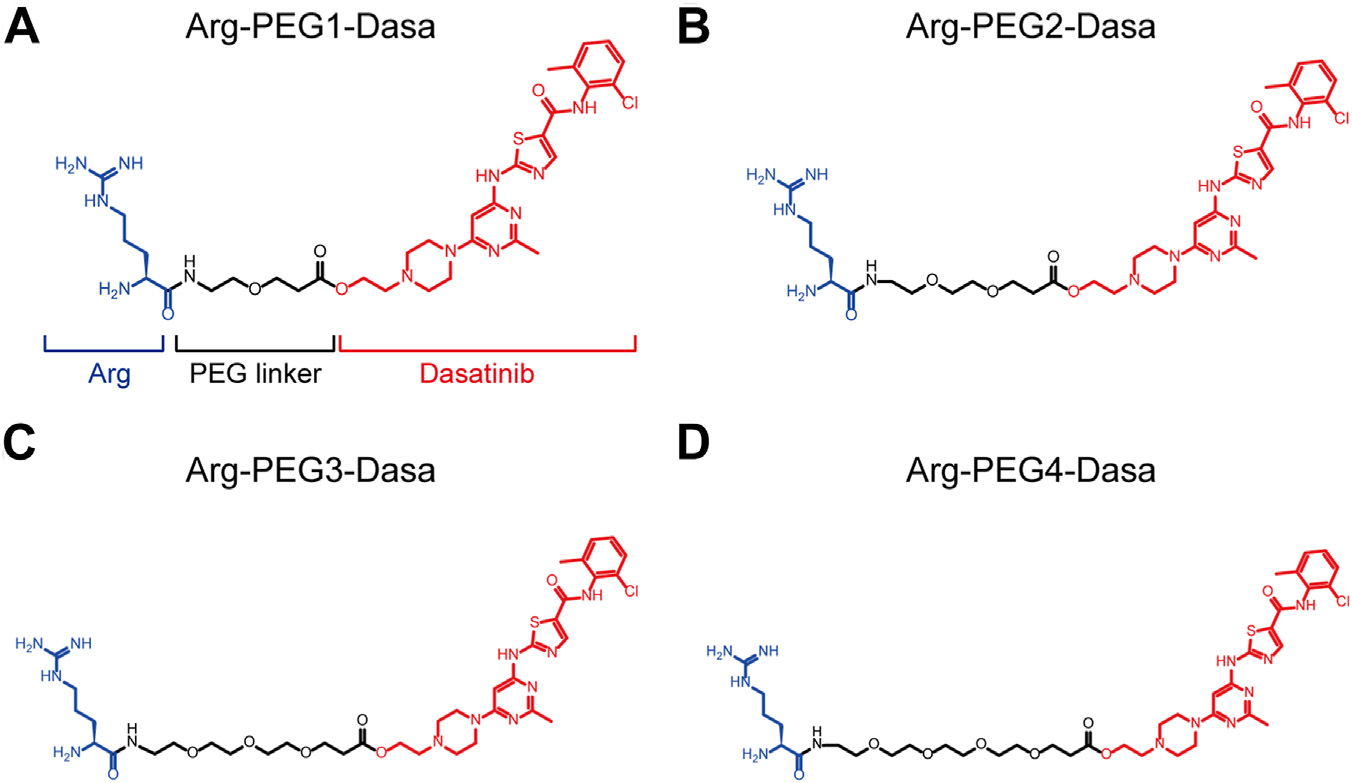
**Chemical structures of the BCR–ABL targeting PROTACs designed.***A*–*D*, schematic of Arg-PEG (1–4)-Dasa with different numbers of PEG as the linker. The synthetic procedures of these compounds are described in the supporting information. ABL, Abelson proto-oncogene; BCR, breakpoint cluster region; Dasa, dasatinib; PROTAC, proteolysis-targeting chimera.

The N-end rule proteolytic pathway is mainly determined by the presence of a destabilizing amino acid at the N terminus of the protein (20, 22). To construct BCR–ABL-targeting PROTACs, we selected two basic residues, Arg and Lys, and two hydrophobic residues, aliphatic Leu and aromatic Phe, as the beginning of the PROTAC to recruit the UBR family E3 Ub ligase. The first set of PROTACs synthesized adopted Arg, a strong destabilizing residue, as the degradation signal (Fig. 1). PEG is employed as the middle linker because of its excellent solubility, biocompatibility, and safety (10, 11, 12, 13). As the length of the linker is often an issue for PROTAC and hard to predict without knowing the dynamic association among E3-PROTAC-target *in vivo*, varying numbers of PEG (1–4) were assessed for their effectiveness (Fig. 1). Dasa was chosen as the warhead to target BCR–ABL (Fig. 1) as Dasa, but not imatinib, was previously found to be adaptable for PROTACs (23).

### Biological evaluation of the BCR–ABL–targeting PROTACs *in vitro*

**Figure 2:**
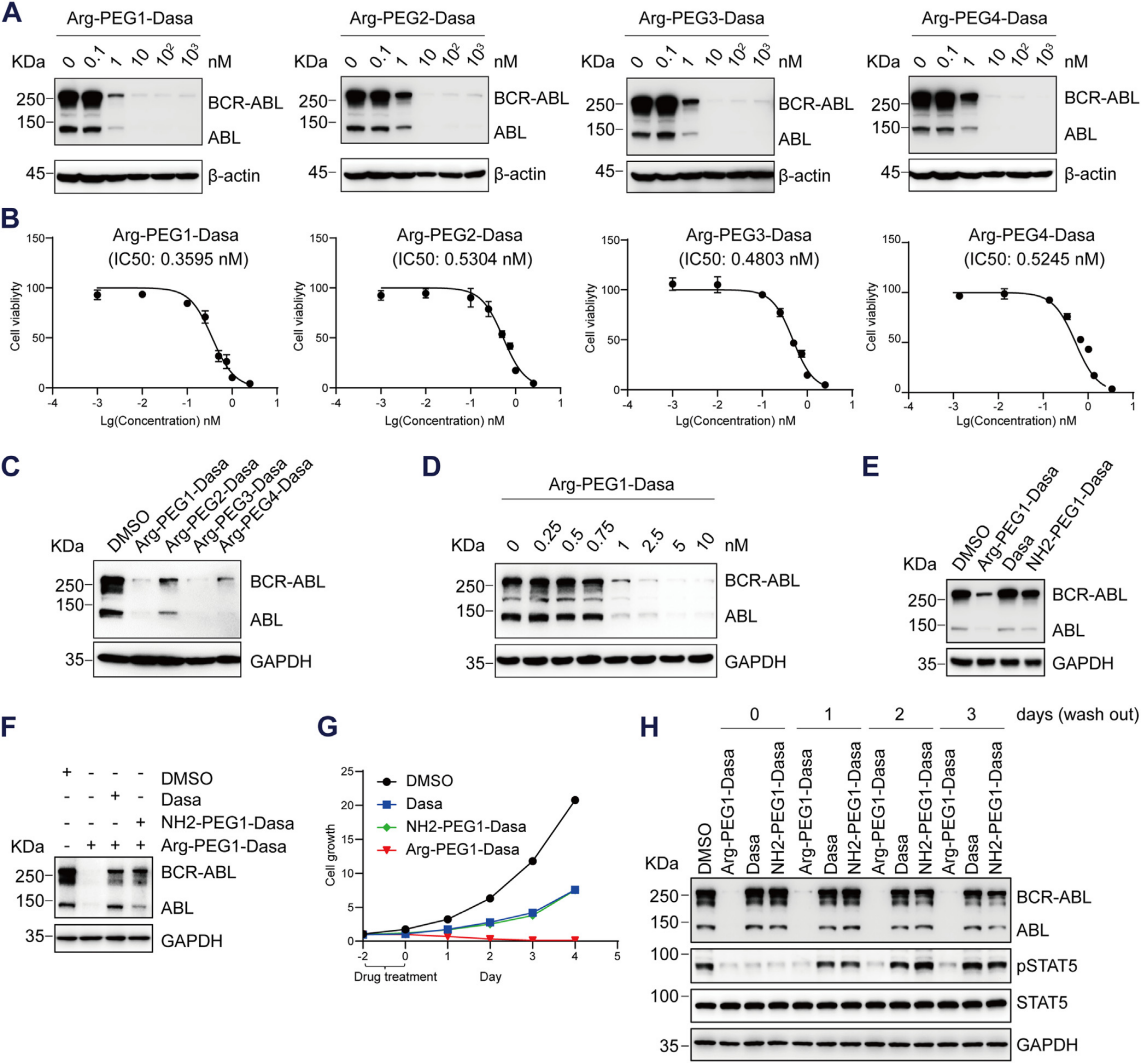
**Arg-PEG (1–4)-Dasa induced the degradation of BCR–ABL through the proteasome.***A*, K562 cells were incubated with Arg-PEG (1–4)-Dasa bearing different numbers of PEG linker at indicated concentration for 48 h. The expression of various proteins indicated was determined by immunoblotting. *B*, K562 cell viability was examined using CCK-8 assays after the treatment with Arg-PEG (1–4)-Dasa for 48 h at various concentrations (0, 0.01, 0.1, 0.25, 0.5, 0.75, 1, and 2.5 nM). *C*, immunoblot analysis of the indicated proteins in K562 cells treated with various compounds at 1 nM for 48 h. *D*, immunoblot analysis of the proteins indicated in K562 cells treated with gradually increasing doses of Arg-PEG1-Dasa for 48 h. *E*, immunoblot analysis of the indicated proteins in K562 cells treated with DMSO, Arg-PEG1-Dasa, Dasa, or NH2-PEG1-Dasa at 1 nM for 48 h. *F*, immunoblot analysis of the indicated proteins in K562 cells pretreated with Dasa or NH2-PEG1-Dasa (1 nM) for 2 h followed by Arg-PEG1-Dasa treatment at 1 nM for 48 h. *G* and *H*, K562 cells were treated with indicated compounds at 1 nM for 2 days, following washout and incubation compound-free medium. *G*, cell growth was detected using CCK-8 assays at the indicated times. *H*, the protein level indicated was tested with immunoblot. ABL, Abelson proto-oncogene; BCR, breakpoint cluster region; CCK-8, Cell Counting Kit-8; Dasa, dasatinib; DMSO, dimethyl sufoxide.

The ability of Arg-PEGn-Dasa PROTACs to induce BCR–ABL degradation was assessed in human CML K562 cells bearing BCR–ABL. Treatment with all four compounds resulted in an apparent downregulation of BCR–ABL protein in K562 cells at 1 nM and elicited a maximal reduction at 10 nM (Fig. 2*A*). Interestingly, the linker with one PEG appeared to be the most effective in tuning down BCR–ABL level. We next evaluated the impact of Arg-PEGn-Dasa on cell proliferation in K562 cells using Cell Counting Kit-8 (CCK-8) assay. K562 cells were treated with the four PROTAC compounds at different concentrations for 48 h. The results showed that the four PROTACs inhibited the proliferation of K562 cells with IC_50_ values ranging from 0.3595 to 0.5304 nM (Fig. 2*B*). Consistent with the degradation data (Fig. 2*A*), the compound with one PEG linker exhibited the best antiproliferation effect (Fig. 2*B*). We directly compared the BCR–ABL reduction by the four compounds side by side (Fig. 2*C*). In line with the aforementioned data (Fig. 2*A*), the compound with one PEG linker was the most efficient in reducing BCR–ABL (Fig. 2*C*). Based on compound size, proteolytic activity, and cellular activity, the Arg-PEG1-Dasa PROTAC with one PEG linker was selected for further analysis. To delineate the effective concentration of Arg-PEG1-Dasa on BCR–ABL degradation, the K562 cells were treated with Arg-PEG1-Dasa at various concentrations from 0 to 10 nM for 48 h. Arg-PEG1-Dasa potently degraded BCR–ABL in K562 cells with a DC_50_ value (the drug concentration that degrades 50% protein) of 0.85 nM and attained 98.8% reduction at ∼5 nM (Fig. 2*D*).

For controls, we also examined the activity of the PROTAC without the single amino acid (an intermediate product NH2-PEG1-Dasa, Fig. S1*A*) or Dasa alone on cell viability of K562 cells for 48 h. Dasa showed IC_50_ values at 0.103 nM (Fig. S1*B*). NH2-PEG1-Dasa inhibited the proliferation of the K562 cells with IC_50_ values at 0.1548 nM (Fig. S1*C*), which was slightly lower than Arg-PEG1-Dasa with an IC_50_ value of 0.3595 nM under continuous exposure to the drug. However, Dasa or NH2-PEG1-Dasa alone failed to reduce BCR–ABL protein levels (Fig. 2*E*). Moreover, the ability of Arg-PEG1-Dasa–mediated degradation of BCR–ABL was mitigated by cotreatment with excess Dasa or NH2-PEG1-Dasa in the competition assay (Fig. 2*F*). One advantage of PROTACs *versus* traditional inhibitors is that only the transient drug–target interaction is needed for its target suppression. Hence, the cell growth was monitored following the pretreatment with dimethyl sulfoxide (DMSO), Dasa, NH2-PEG1-Dasa, or Arg-PEG1-Dasa for 48 h, and then the cells were rinsed with PBS to wash out the drugs and incubated in drug-free medium for additional 1 to 4 days. We found that Arg-PEG1-Dasa showed sustained growth inhibition following drug removal, whereas K562 cell proliferation gradually resumed after the removal of Dasa and NH2-PEG1-Dasa (Fig. 2*G*). Consistently, the protein level of BCR–ABL and its downstream signaling effort of pSTAT5 remained markedly diminished after the Arg-PEG1-Dasa withdrawal, whereas STAT5 phosphorylation was gradually recovered following the removal of Dasa and NH2-PEG1-Dasa (Fig. 2*H*), suggesting that PROTAC has a longer lasting effect than traditional inhibitors.

### BCR–ABL–targeting PROTAC degrades BCR–ABL through the proteasome

**Figure 3:**
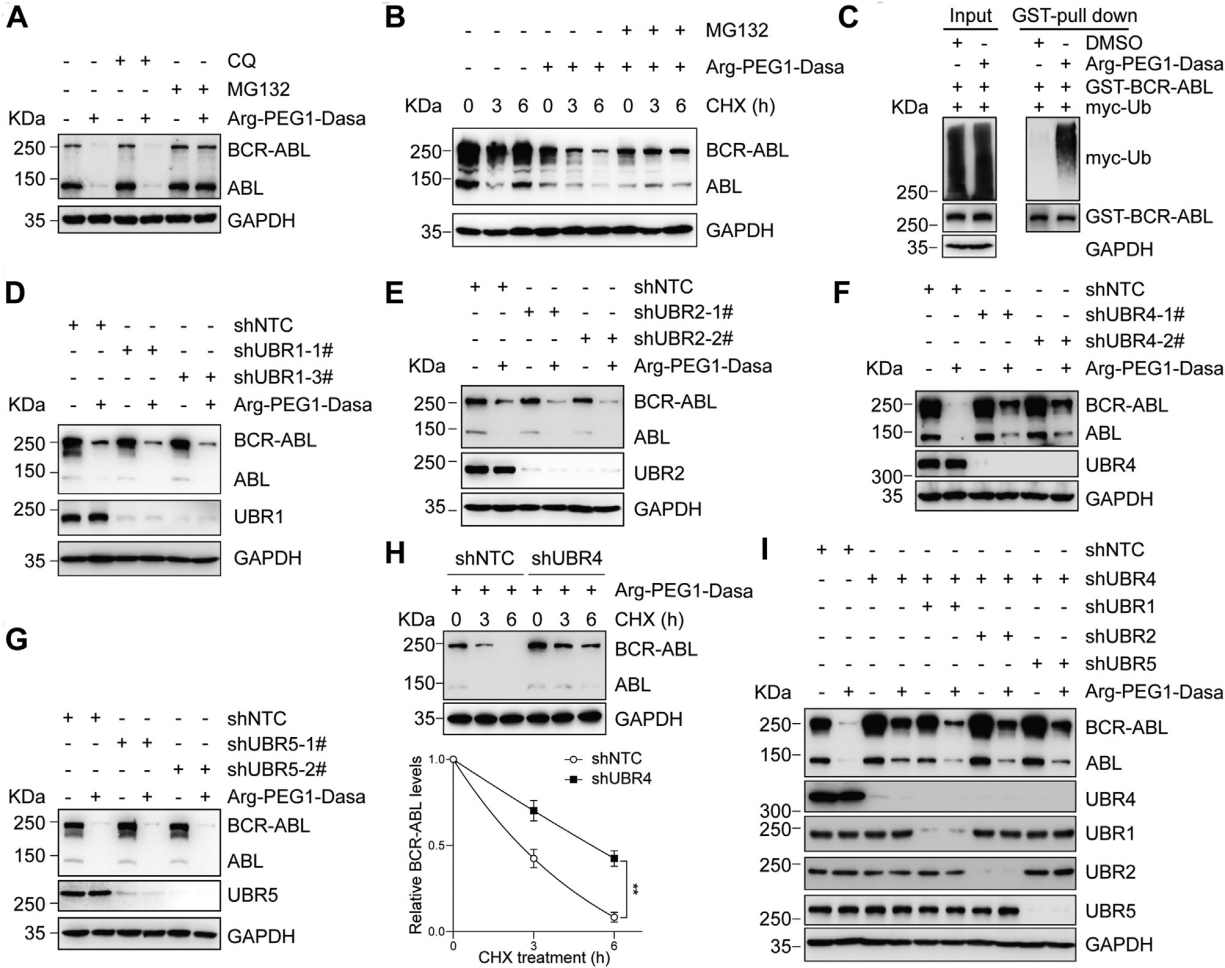
**Arg-PEG1-Dasa mediated the degradation of BCR–ABL *via* the ubiquitin–proteasome pathway.***A*, K562 cells were treated with MG132 (10 μM) or CQ (25 μM) in the absence or the presence of Arg-PEG1-Dasa at 1 nM for 48 h. *B*, K562 cells were treated with 2.5 nM Arg-PEG1-Dasa for 32 h and then incubated with DMSO or MG132 (10 μM). BCR–ABL stability was detected after the addition of cycloheximide (CHX) at the indicated time points using immunoblot. *C*, HEK293T cells bearing myc-Ub and GST-BCR–ABL were treated with DMSO or Arg-PEG1-Dasa for 48 h. Ubiquitination of GST-BCR–ABL was examined by denaturing immunoprecipitation (IP) with anti-Myc antibody. *D*–*G*, K562 cells stably expressing indicated shRNA were treated with DMSO or Arg-PEG1-Dasa for 48 h. The protein level indicated was monitored with immunoblot. *H*, K562 cells stably expressing control or UBR4 shRNA were treated with Arg-PEG1-Dasa for 32 h, followed by incubating with CHX at the indicated time points. The protein level was assayed by immunoblots (*upper panel*). The intensity of BCR–ABL bands was quantified (means ± SD in three independent experiments, ∗∗*p* < 0.01, two-tailed Student's *t* test, *lower panel*). *I*, K562 cells stably expressing indicated shRNA were treated with Arg-PEG1-Dasa for 48 h. ABL, Abelson proto-oncogene; BCR, breakpoint cluster region; CQ, chloroquine; Dasa, dasatinib; DMSO, dimethyl sulfoxide; GST, glutathione-*S*-transferase; HEK293T, human embryonic kidney cell line; Ub, ubiquitin.

To determine the degradation pathway of BCR–ABL induced by PROTAC, we added lysosome acidification inhibitor chloroquine or proteasome inhibitor MG132 into Arg-PEG1-Dasa–treated K562 cells. The result showed that MG132, not chloroquine, blocked Arg-PEG1-Dasa–mediated degradation of BCR–ABL, suggesting that Arg-PEG1-Dasa causes degradation of BCR–ABL in proteasome-dependent manner (Fig. 3*A*). To ascertain that Arg-PEG1-Dasa induced BCR–ABL turnover, we monitored the degradation kinetics of BCR–ABL with or without the PROTAC treatment. K562 cells were treated with cycloheximide to block protein synthesis and sampled at various time points for BCR–ABL levels. BCR–ABL protein was quickly reduced in the Arg-PEG1-Dasa–treated cells but remained largely stable in DMSO-treated cells (Fig. 3*B*). To further evaluate the involvement of the proteasome in PROTAC-induced degradation, we also added the proteasome inhibitor MG132 in the reaction. The Arg-PEG1-Dasa–induced BCR–ABL turnover was compromised by MG132 (Fig. 3*B*), indicating that BCR–ABL turnover requires the proteasome. We next sought to investigate the Arg-PEG1-Dasa–induced ubiquitination of BCR–ABL. When human embryonic kidney 293T cells coexpressing glutathione-*S*-transferase-BCR–ABL and myc-Ub were treated with DMSO or Arg-PEG1-Dasa, only the Arg-PEG1-Dasa–treated cells displayed the ubiquitination of immunoprecipitated BCR–ABL (Fig. 3*C*). Collectively, these experiments suggest that Arg-PEG1-Dasa induced BCR–ABL degradation mainly through the Ub-dependent proteasome system.

It has been reported that mammalian E3 Ub ligases including UBR1, UBR2, UBR4, and UBR5 (N-recognins) may mediate the proteolysis of the substrates with destabilizing N-terminal residues (N-degrons) *via* N-end rule pathway (20, 24). We assessed which N-recognin participates in the PROTAC-mediated degradation of BCR–ABL. To this end, we silenced UBR1, UBR2, UBR4, and UBR5 in K562 cells, respectively, then treated these cells with Arg-PEG1-Dasa for 48 h. We found that only knockdown of UBR4 hindered the Arg-PEG1-Dasa–induced reduction of BCR–ABL partially (Fig. 3, *D*–*G*). To further test the effect of UBR4 on Arg-PEG1-Dasa–induced BCR–ABL turnover, we monitored the degradation kinetics of BCR–ABL in UBR4 knockdown or control cells upon the Arg-PEG1-Dasa treatment. BCR–ABL protein was stabilized after UBR4 knockdown (Fig. 3*H*). Furthermore, as N-recognins are functionally redundant (20, 24, 25), we wondered whether UBR4 may work with another N-recognin collaboratively to promote PROTAC-mediated BCR–ABL degradation. However, the double knockdowns of UBR4 with UBR1, UBR2, or UBR5 was not able to further impede Arg-PEG1-Dasa–induced BCR–ABL degradation (Fig. 3*I*).

### The N-end rule–based PROTACs modulate the degradation rate of BCR–ABL protein

**Figure 4:**
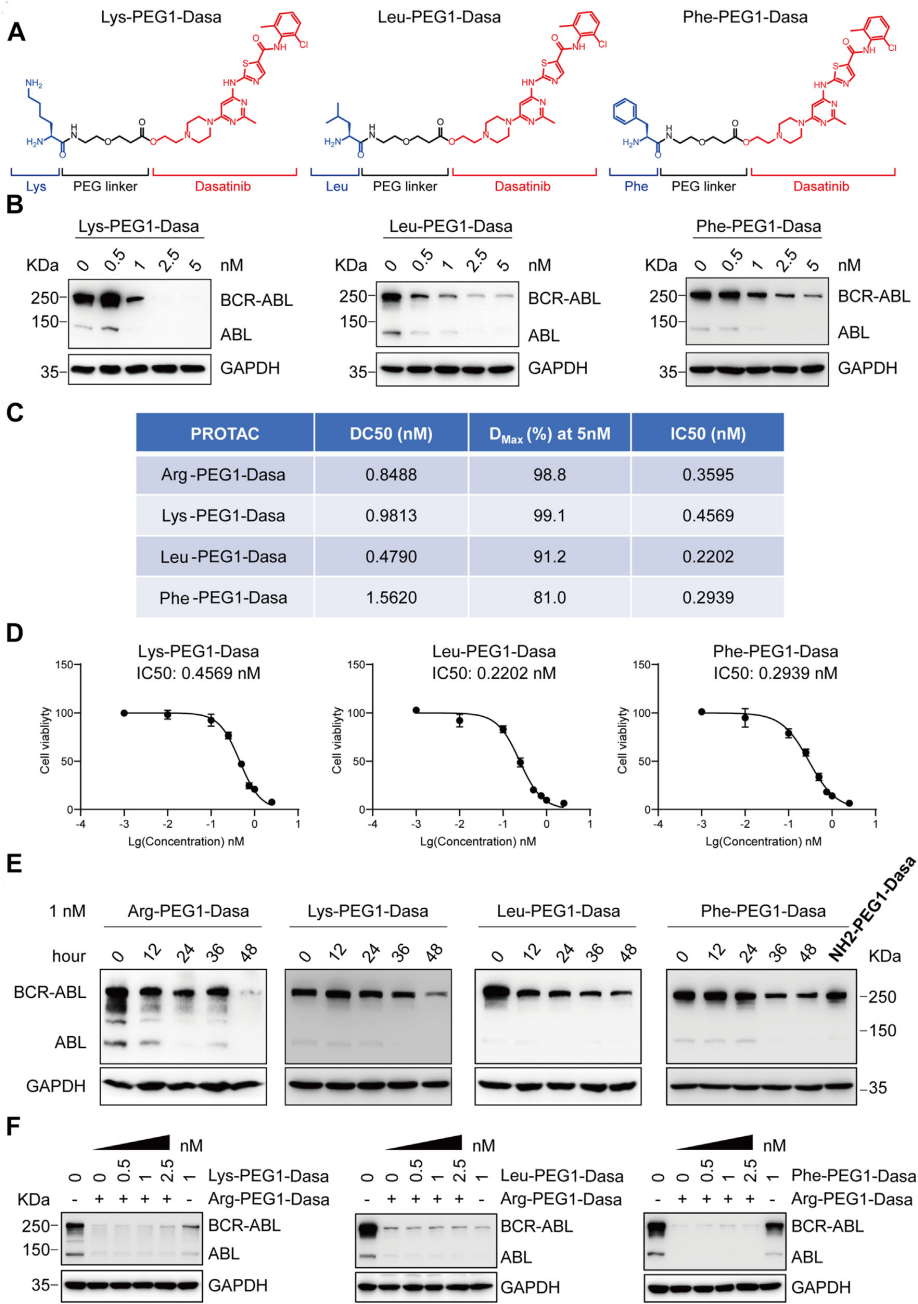
**Analysis of BCR–ABL targeting PROTACs with different amino acids.***A*, chemical structures of Lys-PEG1-Dasa, Leu-PEG1-Dasa, and Phe-PEG1-Dasa. *B*, K562 cells were incubated with Lys-PEG1-Dasa, Leu-PEG1-Dasa, and Phe-PEG1-Dasa separately at the indicated concentrations for 48 h. The expression of various proteins was determined by immunoblotting. *C*, summary of DC_50_ (the drug concentration that leads to 50% protein degradation), *D*_Max_ (the maximum degradation percentage in comparison with control), and IC_50_ (the half maximal inhibitory concentration) values of the PROTACs. *D*, K562 cell viability was determined using CCK-8 assays upon the treatment with Lys-PEG1-Dasa, Leu-PEG1-Dasa, or Phe-PEG1-Dasa for 48 h at varying concentration (0, 0.01, 0.1, 0.25, 0.5, 0.75, 1, and 2.5 nM). *E*, immunoblot analysis of various proteins in K562 cells treated with Arg-PEG1-Dasa, Lys-PEG1-Dasa, Leu-PEG1-Dasa, or Phe-PEG1-Dasa at 1 nM for the indicated time. Intermediate product NH2-PEG1-Dasa (NH2), also mixed with K562 cells at 1 nM for 48 h, is used as a control. Structure of NH2-PEG1-Dasa lacking an amino acid is depicted in supporting information. *F*, combined effects of two PROTACs on BCR–ABL degradation in K562 cells. ABL, Abelson proto-oncogene; BCR, breakpoint cluster region; CCK-8, Cell-Counting Kit 8; Dasa, dasatinib; PROTAC, proteolysis-targeting chimera.

Eight primary destabilizing N-terminal residues determine the protein turnover through N-end rule pathway (20). The rate of protein degradation based on the N-end rule could be regulated by the strength of the interaction between different amino acids and the E3 Ub ligases. To explore whether the degradation speed may be influenced by different amino acids, we conjugated three amino acids with different properties (Lys, Leu, or Phe) to Dasa with one PEG linker, producing the PROTACs Lys-PEG1-Dasa, Leu-PEG1-Dasa, and Phe-PEG1-Dasa (Fig. 4*A*). We found that these three compounds exhibited efficient but different rate of BCR–ABL reduction in K562 cells and achieved 81.0 to 99.1% decrease at around 5 nM (Fig. 4, *B* and *C*). We then carried out the CCK-8 assay to evaluate the growth inhibitory effect of three PROTACs. These three compounds have IC_50_ value in between 0.2 and 0.5 nM in K562 cells (Fig. 4*D*), suggesting that the N-end rule–based PROTACs tune down BCR–ABL efficiently and confer a potent antiproliferation activity in K562 cells (Fig. 4, *B*–*D*).

To further assess the effectiveness of these PROTACs on BCR–ABL degradation, K562 cells were incubated with all four PROTACs at 1 nM for different length of time. Two basic amino acids Arg and Lys-based PROTACs, induced the apparent reduction of BCR–ABL proteins at 48 h. Whereas the aliphatic amino acid Leu-based PROTAC led to the decrease of BCR–ABL at 12 h and the aromatic amino acid Phe-based PROTAC began to show BCR–ABL reduction at 36 h (Fig. 4*E*). We also treated K562 cells with an intermediate product (NH2-PEG1-Dasa) as a negative control at 1 nM for 48 h. The result revealed that NH2-PEG1-Dasa failed to trigger BCR–ABL reduction significantly (Fig. 4*E*, *last lane*). Collectively, our data suggest that the N-end rule–based PROTACs can reduce target concentration and inhibit cell growth potently, which can also be modulated simply with different amino acids (Fig. 4, *B*–*E*).

We also evaluated whether either Lys-, Leu-, or Phe-PEG1-Dasa is synergistic with Arg-PEG1-Dasa in degradation of BCR–ABL, and we mixed Arg-PEG1-Dasa with varying doses of Lys-, Leu-, or Phe-PEG1-Dasa (Fig. 4*F*). We found that the degradation of BCR–ABL was not further enhanced when Arg-PEG1-Dasa was used in combination with either Lys-, Leu-, or Phe-PEG1-Dasa, which may be due to the fact that Arg-PEG1-Dasa itself is very effective.

### Combined knockdown of UBR1, UBR2, and UBR4 weakened degradation of BCR–ABL mediated by the N-end rule–based PROTACs

**Figure 5:**
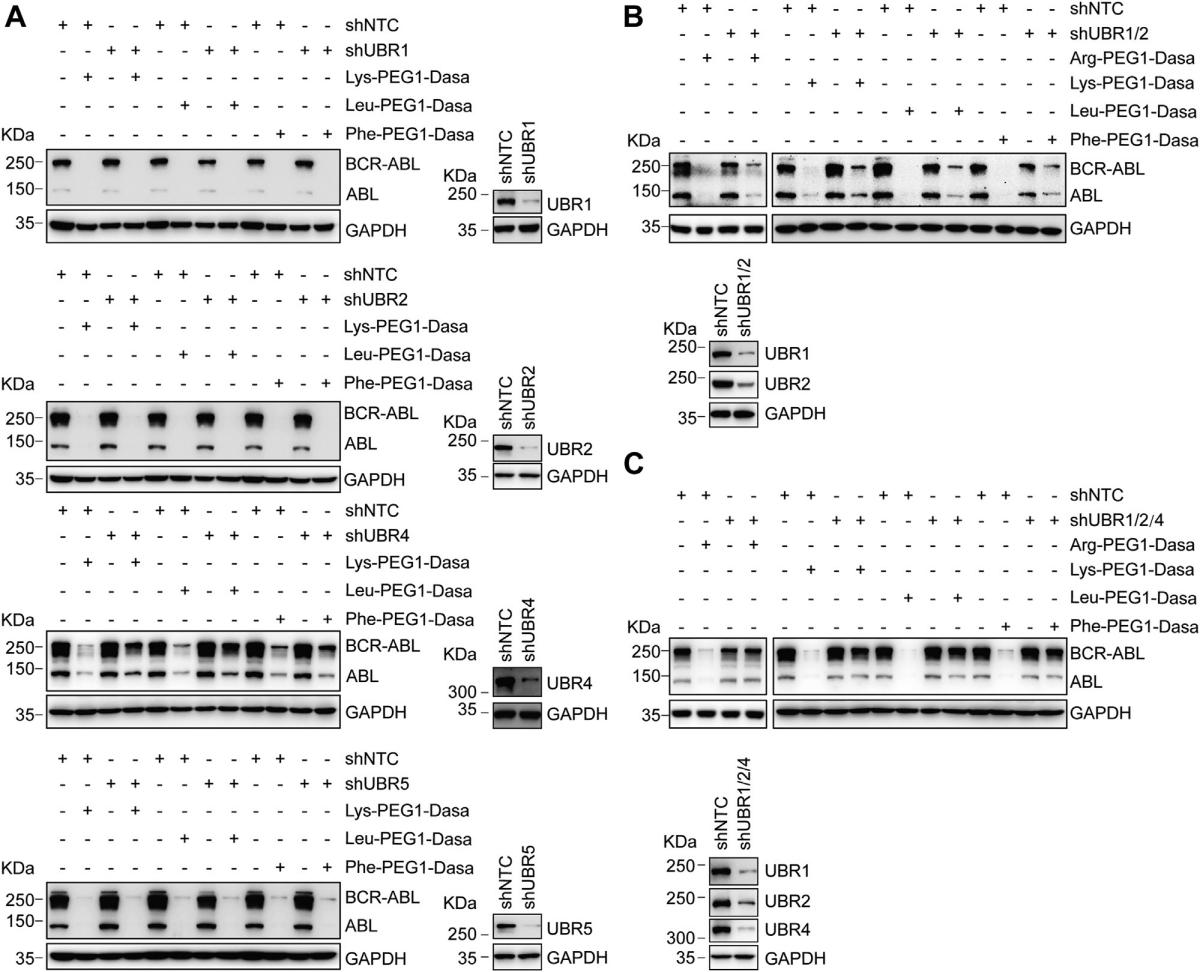
**Ubiquitin ligases UBR1, UBR2, and UBR4 participate in the N-end rule–based PROTAC-induced degradation of BCR–ABL.***A*, K562 cells stably expressing indicated shRNA were treated with DMSO or Lys-, Leu-, or Phe-PEG1-Dasa for 48 h. The expression level of various proteins was monitored with immunoblots. *B*, control or the double knockdown of UBR1 and UBR2 in K562 cells was treated with DMSO or Arg-, Lys-, Leu-, or Phe-PEG1-Dasa for 48 h. Various proteins indicated were detected by immunoblots. *C*, effects of the triple knockdown of UBR1, UBR2, and UBR4 E3s on PROTAC-induced BCR–ABL degradation in K562 cells. ABL, Abelson proto-oncogene; BCR, breakpoint cluster region; Dasa, dasatinib; DMSO, dimethyl sulfoxide; PROTAC, proteolysis-targeting chimera.

To delineate which N-recognin E3 participates in the PROTAC-mediated degradation of BCR–ABL, we silenced UBR1, UBR2, UBR4, and UBR5 in K562 cells, respectively (Fig. 5). As shown in Figure 5*A*, only UBR4 knockdown impeded the Lys-, Leu-, or Phe-PEG1-Dasa–triggered reduction of BCR–ABL partially, which is similar to its effects in the Arg-PEG1-Dasa–treated cells (Fig. 3). Given functional redundancy of UBR1 and UBR2, we knocked down both UBR1 and UBR2 in K562 cells and then treated these cells with various PROTACs. Silencing both UBR1 and UBR2 slightly affects the Arg-, Lys-, Leu-, or Phe-PEG1-Dasa–triggered reduction of BCR–ABL (Fig. 5*B*). Since single knockdown of UBR4 or, to a less extend, the double knockdown of UBR1 and UBR2 seems to partially disrupt PROTAC-induced BCR–ABL degradation, we wonder the effect of combinatorial silence of the E3 ligases UBR1, UBR2, and UBR4. We found that the triple knockdown of UBR1, UBR2, and UBR4 largely blocked N-end rule–based PROTAC-induced BCR–ABL (Fig. 5*C*).

### Antitumor efficacy of N-end rule–based PROTAC *in vivo*

**Figure 6:**
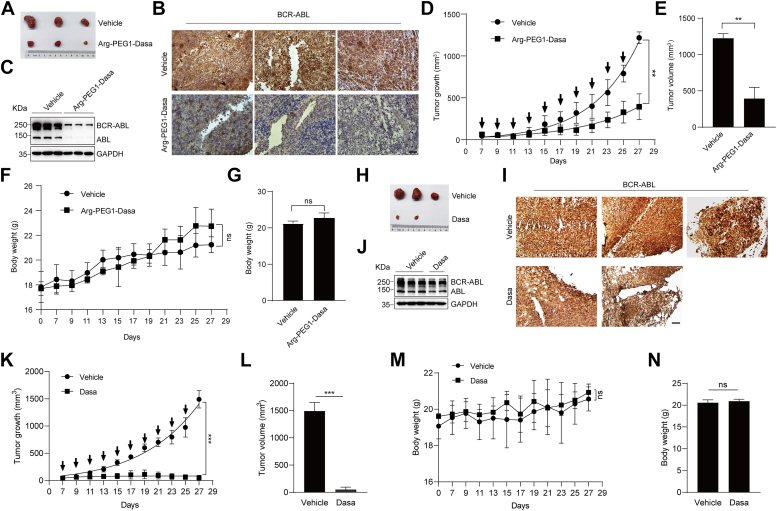
**Arg-PEG1-Dasa inhibits tumor growth *in vivo*.***A*, K562 cells were inoculated into the *right flank* of 4-week-old female nude mice. The mice were treated with vehicle or Arg-PEG1-Dasa (10 mg/kg, i.p.) starting from day 7 post-transplantation. The drugs were administered every other day for a total of 10 doses. Excised tumors at day 27 from each group are photographed. *B*, representative immunohistochemical labeling of BCR–ABL in tumor sections of mice from *A*. The nuclei were counterstained with hematoxylin (*blue*). Scale bars represent 50 μm. *C*, immunoblot analysis of the BCR–ABL protein level in tumor tissue of mice from *A*. *D*, the graph depicts the mean tumor growth of mice in *A*, which received the indicated treatment on the days marked by the *black arrows*. *E*, the bar graphs show the mean ± SD of the primary tumor volume from mice in *A*. *F*, the graph depicts the mean body weight of mice in *A*, which received administration described in *E*. *G*, the bar graphs indicate the mean ± SD of body weight of mice at the endpoint of treatment for each group. *H*, K562 cells were inoculated into the *right flank* of 4-week-old female nude mice. The mice were treated with vehicle or Dasa (10 mg/kg, i.p.) starting from day 7 post-transplantation. The drugs were administered every other day for a total of 10 doses. Excised tumors at day 27 from each of group are photographed. *I*, representative immunohistochemical labeling of BCR–ABL in tumor sections of mice from *H*. The nuclei were counterstained with hematoxylin *(blue*). Scale bars represent 50 μm. *J*, immunoblot analysis of the BCR–ABL protein level in tumor tissue of mice from *H*. *K*, the graph depicts the mean tumor growth of mice in *H*, which received the indicated treatment on the days marked by the *black arrows*. *L*, the bar graphs show the mean ± SD of the primary tumor volume from mice in *K*. *M*, the graph depicts the mean body weight of mice in *H*, which received administration described in *K*. *N*, the bar graphs indicate the mean ± SD of body weight of mice at the endpoint of treatment for each group. For *D*–*G* and *K*–*N*, ∗∗*p* < 0.01, ∗∗∗*p* < 0.001, two-tailed Student’s *t* test. ABL, Abelson proto-oncogene; BCR, breakpoint cluster region; Dasa, dasatinib; ns, no statistical significance.

To evaluate the effects of the N-end rule–based PROTAC on tumor growth *in vivo*, we set up a K562 CML xenograft mouse model. K562 cells were subcutaneously implanted into the right flank of female nude mice. When the tumors reached a palpable volume of about 50 to 80 mm^3^ at day 7, the mice bearing K562 tumors were then divided into two groups randomly and administered every other day with either vehicle or Arg-PEG1-Dasa (10 mg/kg) for total 10 doses by intraperitoneal injection. Tumor volumes were measured at indicated time with calipers. When sacrificed on day 27, the tumors were harvested and photographed (Fig. 6*A*). The amount of BCR–ABL protein *in vivo* upon Arg-PEG1-Dasa treatment was examined in K562 xenograft mouse model using the immunohistochemistry (Fig. 6*B*) and immunoblot (Fig. 6*C*). The results manifested that the tumor size and BCR–ABL levels were significantly downregulated in the Arg-PEG1-Dasa–treated groups (Fig. 6, *A*–*C*). Moreover, the Arg-PEG1-Dasa intervention dramatically impeded tumor growth compared with vehicle-treated control (Fig. 6, *D* and *E*). Importantly, Arg-PEG1-Dasa did not significantly alter the body weight of the mice, suggesting that the dosage and therapeutic regimen were well tolerated in mice (Fig. 6, *F* and *G*). In addition, Dasa only was also similarly administered and exhibited potent tumor growth inhibition *in vivo* without significant loss of body weight and failed to reduce protein levels of BCR–ABL, as reported in previous studies (26) (Fig. 6, *H*–*N*). Further studies are needed to optimize PROTAC or administration strategy to achieve better therapeutic effects.

## Discussion

PROTAC technology has garnered considerable attention and interests in the drug discovery industry because of its advantages in targeting undruggable proteins, recyclability, and overcoming drug resistance (13, 27). However, this approach has also manifested several pressing shortcomings such as poor cell permeability because of the larger molecular weight of PROTAC compounds made of three components, limited number of E3 ligases currently available for PROTACs, and the lack of degradation speed control (13, 15, 18, 21). In this study, we demonstrate the utility of the N-end rule–based PROTACs in BCR–ABL degradation, which can be further adjusted *via* different amino acids (Fig. 4), a unique feature of our approach. Importantly, the PROTACs designed effectively inhibit cancer cell proliferation in the nanomolar range *in vitro* (Figs. 2 and 4) and tumor growth *in vivo* (Fig. 6).

Despite more than 600 E3 ligases in humans, only a handful of E3 ligases (*e.g.*, CRBN, VHL, IAPs, and MDM2) have been adapted for PROTAC (13, 15, 16). Thus, the application of PROTAC is limited to these selected few E3 ligases with restricted tissue- or organ-specific expression and their ligands. The N-end rule pathway, the very first Ub-mediated proteolytic route identified (20, 22, 24), is highly conserved from yeast to human. N-terminal degradation signal sequences (N-degrons) include type 1 (basic amino acids: Arg, Lys, and His) and type 2 (bulky hydrophobic amino acids: Leu, Ile, Phe, Tyr, and Trp) destabilizing residues. Mammalian E3 Ub ligases including UBR1, UBR2, UBR4, and UBR5 are able to recognize these N-degrons to promote substrate ubiquitylation and degradation *via* Ub–proteasome system (20, 24). It is reported that UBR Ub ligases are broadly expressed in many tissues and organs (20, 24, 28, 29). We have previously demonstrated as a proof of principle that Arg- and His-derived PROTACs can trigger the degradation of estrogen-related receptor α in an N-end rule and proteasome-dependent manner (21). However, there are several caveats with the earlier study (21): (1) The effective doses of the PROTACs were quite high, ∼5 to 10 μM; (2) The experimental setting with estrogen-related receptor α is not necessarily clinically relevant; (3) It was not obvious that distinct amino acids elicit different effects as Arg and His are both basic residues and generated similar outcomes; (4) last, but not the least, those PROTACs were not subjected to *in vivo* analysis for their efficiency (21). This is an important issue as it is not given that the success of these compounds at cellular level can be easily duplicated *in vivo* (*e.g.*, mouse), a step that took nearly 15 years for the validation of PROTAC (10), leading to ensuing “gold rush” (10, 12, 13). Using distinct amino acids in the background of BCR–ABL–triggered CML, this study illustrates the efficacy of the N-end rule–based PROTAC in disease relevant settings *in vitro* and *in vivo* for the first time.

We have adapted four of eight primary residues in our PROTACs here with different degradation potency (Fig. 3). Basic amino acids, Arg- and Lys-based PROTACs, exhibit similar degradation rate of BCR–ABL with DC_50_ ∼0.85 nM and 0.98 nM, respectively, suggesting that amino acids with the same properties might lead to comparable substrate turnover. Hydrophobic residues appear to show bigger differences as the DC_50_ for aliphatic amino acid Leu and aromatic residue Phe are 0.48 nM and 1.56 nM, respectively. Further modifications of these destabilizing residues could modulate their affinity for the four mammalian UBR E3 ligases involved in the N-end rule pathway, possibly generating a broader range of degradation capacity. Whereas type 1 basic residues are recognized by the UBR box domain of the E3 Ub ligases (*i.e.*, UBR1, UBR2, UBR4, and UBR5), type 2 hydrophobic residues bind to the N domain of UBR1 and UBR2 (20, 24). Knockdown of UBR4 or UBR1 and UBR2 partially impaired N-end rule–based PROTAC-induced BCR–ABL downregulation. Previous study shows that the combined deletion of UBR1, UBR2, and UBR4 may block degradation of substrates with type II destabilizing residues and partially obstructs degradation of substrates with type I destabilizing residues by the N-end rule pathway (30). Consistently, our data showed that combinatorial knockdown of UBR1, UBR2, and UBR4 largely blocked N-end rule–based PROTAC-induced BCR–ABL turnover (Fig. 5). However, residual degradation of BCR–ABL remains in the cells deficient for UBR1, UBR2, and UBR4 E3s, suggesting that there may be additional Ub ligase E3s involved. Given the functional redundancy of the UBR box E3 ligases in mammals and distinction of N-degrons (20, 24, 25), we speculate that multiple E3 N-recognins cooperate to mediate the function of N-end rule–based PROTAC. The identity of specific E3 ligases involved in the action of these PROTACs requires further investigation.

It has been found that the length and flexibility of linkers markedly impact the degradation effect of PROTACs (13, 15, 31). It is hard to model the *in vivo* productive association of the E3–PROTAC–target complex. The PROTAC linkers are often determined through trials and errors (13, 15, 31). In an excellent analysis of BCR–ABL targeting PROTACs, Crews *et al.* found only specific combination of E3 (*i.e.*, CRBN), linker (*i.e.*, 6-2-2-6), and target ligand (*i.e.*, Dasa) works well (23). The effective linker 6-2-2-6 contains three hydrophilic PEGs with the extension of hydrophobic moieties on both sides (23). The PEG-based linker has been frequently used in PROTACs because of its excellent solubility, biocompatibility, as well as stability and safety in humans (32, 33, 34). To find the optimal length of the linker, we designed the PROTACs with varying number (1–4) of PEGs. Interestingly, the Arg-derived PROTAC with only one PEG achieves the best degradation efficacy on BCR–ABL. As the linker for UBR ligand (*e.g.*, Arg, Leu) and Dasa appears to be shorter and much easier to find than the linker for CRBN ligand and Dasa (23), it is tempting to speculate that the association between the UBR E3 Ub ligase and its ligand (amino acids like Arg, Leu, and Lys) may be more flexible and conducive for the PROTAC application. It is worth to note that our single amino acid–based PROTACs exhibit a lower DC_50_ (<1 nM) and IC_50_ (<0.5 nM) values compared with many previously reported BCR–ABL–targeting PROTACs with DC_50_ at the range of 8.5 nM to 30 μM and IC_50_ from 3.4 nM to 3.4 μM (26, 35, 36).

Cell growth and proliferation require delicate balance of protein synthesis and degradation. Many key cellular regulators need to be kept in the right range since too much or little of these proteins could turn to be harmful in some cases. Therefore, an ideal PROTAC is not the one that could destroy the target the most but the one that keeps the target in the right range for normal cellular function. For this reason, it is imperative to design PROTACs that could easily generate a range of target protein concentration. There are some modular designs of PROTACs that can be turned on or off by light, oxygen, chemicals, or other changes, which may bring undesired physiological alterations with known or unknown consequences (37, 38, 39, 40). We demonstrate that the N-end rule–based PROTACs with distinct amino acids (Arg, Lys, Leu, and Phe) easily provide a range of BCR–ABL concentration under the same condition (Fig. 4), and the Arg-PEG1-Dasa can effectively suppress BCR–ABL–induced tumor growth without overt drug toxicity *in vivo* (Fig. 6). Combined, our study highlights the advantages of the N-end rule–based PROTAC, including small size, low concentration, high potency, and easily adjustable, further expanding the toolbox of the E3 ligases and ligands effective for PROTACs in the fight against cancers and other diseases.

Although BCR–ABL TKIs have already displayed excellent efficacy in the treatment of CML, patients need extended or even lifetime TKI therapy, which increases risk of complications and economic burden. The withdrawal of Dasa quickly lead to the rapid recovery of K562 cell growth and BCR–ABL downstream kinase signaling, whereas the use of the N-end rule–based PROTAC showed more durable inhibition of K562 cell growth, BCR–ABL protein, and its downstream kinase signaling under the same condition. Thus, the sustained effect after drug withdraw might be a general advantage of PROTAC, which would benefit CML patients from clinical perspective. Persistent TKI treatment provides time for cells to develop alternative ways to adapt and often results in inevitable secondary mutations and acquired drug resistance. In comparison with traditional occupancy-driven small-molecule drugs that act through sustained association with target protein, PROTACs function *via* transient binding to eliminate target upon contact, which limits the time of drug engagement and may alleviate drug resistance (10, 11, 12, 13).

Acquired mutations may maintain the drug binding but alter protein conformation to bypass the inhibition of the drug (*e.g.*, gefitinib), in which case the PROTACs could still bind and destroy the target. In the cases likes these, the PROTACs could still work against drug-resistant cells. However, the Dasa-resistant mutations (*e.g.*, T315I) are known to disrupt the Dasa binding, in which case the Dasa-based PROTACs would lose its effectiveness. In the cases like Dasa, one would need to employ either different compounds (*e.g.*, ponatinib, olverembatinib, and allosteric inhibitors) or Dasa derivatives that could recognize BCR–ABL T315I mutant (41, 42, 43, 44).

Despite of the focus on and the advancement of PROTACs in recent years, a number of challenging issues remain. For example, whereas some PROTACs appear superior than the ligands without the degradation moiety, some PROTACs are not obviously better than the inhibitors developed earlier at least in some settings (23, 26, 42, 45, 46, 47). In our case, Arg-PEG1-Dasa does not exhibit better antitumor effects than Dasa itself in K562 xenograft model under the same therapeutic regimen (Fig. 6), which is similar to other BCR–ABL–targeting PROTACs reported previously (26, 44, 48). Successful drugs like Dasa and imatinib are well designed, tested, optimized, and refined over the time. With further addition of linkers and the degradation signal, the resulting PROTACs tend to be bigger, over the rule of five commonly known for optimal compound size, and likely alter the solubility, membrane permeability, pharmacokinetics, and the binding affinity for BCR–ABL (10, 11, 12, 13). Nevertheless, with the improved efficacy and strong potential demonstrated for other PROTACs, it is worthwhile to further optimize the N-end rule–based and/or BCR–ABL-targeting PROTACs for better therapeutic effects.

## Experimental procedures

### Reagents

Antibodies used include anti-c-Abl (catalog no.: 2862; 1:1000 dilution), anti-myc (catalog no.: 2040, 1:2000 dilution), and anti-UBR5 (catalog no.: 65344, 1:1000 dilution) from Cell Signaling Technology; anti-GAPDH (catalog no.: HRP-60004, 1:6000 dilution) from Proteintech; anti-UBR1 (catalog no.: sc-515753, 1:1000 dilution), and anti-β-actin (catalog no.: sc-517582, 1:2000 dilution) from Santa Cruz Biotechnology; anti-UBR2 (catalog no.: ab217069, 1:1000 dilution) and anti-UBR4 (catalog no.: ab86738, 1:1000 dilution) from Abcam; anti-pSTAT5 (catalog no.: AP0758, 1:1000 dilution) from IBIAN Technologies; and anti-STAT5 (catalog no.: D220085, 1:2000 dilution) from BBI Life science. MG132 and cycloheximide were obtained from MedChemExpress. Dasa, HATU, DEA, TFA, DIPEA, DMAP, DCC, CH3CN, Boc-Arg (Pbf)-OH, Fmoc-Lys (Boc)-OH, and Fmoc-Phe-OH were purchased from Energy Chemical. Fmoc-Leu-OH was purchased from Aladdin. Fmoc-PEG1-(CH2)-COOH, Fmoc-PEG2-(CH2)-COOH, Fmoc-PEG3-(CH2)-COOH, and Fmoc-PEG4-(CH2)-COOH were purchased from Leyan. Anhydrous dimethylformamide and dichloromethane were purchased from J&K Scientific.

### Cell culture

K562 cell line was purchased from the American Type Culture Collection and maintained in RPMI1640 (Thermo Scientific) plus 10% fetal bovine serum (ExCell Bio) and penicillin–streptomycin (Thermo Scientific). Mycoplasma testing of cell culture was performed routinely using a MycoBlue Mycoplasma Detector Kit (Vazyme).

### PROTAC synthesis and mass spectrum identification

The chemical synthesis procedure and mass spectrum identification of PROTACs are described in the supporting information section.

### PROTAC characterizations

Analytical RP-HPLC was performed on an Agilent 1260 infinity system equipped with a DAD-UV detector using an Agilent Poroshell 120, EC-C18 column (4.6 mm × 100 mm, 2.7 μm). The RP-HPLC gradient was started at 10% of B (CH3CN) and then increased to 100% of B over 20 min (A: 0.1% TFA in water) with a flow rate of 0.5 ml/min. The purity of the compounds used for biological study (>95%) was determined by HPLC. Semipreparative RP-HPLC was performed on the ULTIMAT 3000 instrument (DIONEX). UV absorbance was measured using a photodiode array detector at 220 and 254 nm. The RP-HPLC gradient was started at 10% of B (CH3CN) and then increased to 100% of B over 20 min (A: 0.1% TFA in water). High-resolution mass spectra were measured with an ABI Q-star Elite.

### Immunoblot analysis

Cells were collected and lysed in radioimmunoprecipitation assay buffer (50 mM Tris–HCl [pH 8.0], 1% NP-40, 150 mM NaCl, 0.1% SDS, 0.5% sodium deoxycholate, and 1× complete protease inhibitor cocktail) on ice for 20 min and centrifuged at 15,000 rpm for 10 min at 4 °C to collect the supernatant. After protein concentrations were measured by the bicinchoninic acid protein assay kit (Thermo Scientific), samples with equal amounts of proteins were mixed with loading buffer and denatured by boiling. Protein lysates were resolved by SDS-PAGE and transferred to polyvinylidene fluoride membranes. Then, the membranes were blocked in 5% nonfat milk for 1 h at room temperature, incubated with a primary antibody overnight at 4 °C, and then incubated with horseradish peroxidase–conjugated secondary antibodies for 1 h at room temperature. Subsequently, the membranes were probed with ECL reagent (Millipore), and proteins were visualized by a Tanon-5200 Automatic Chemiluminescence Imaging Analysis System (Tanon).

### Lentiviral production and infection

A lentiviral vector bearing the shRNA of UBR1, UBR2, UBR4, and UBR5, along with a packing vector (psPAX2) and an envelope vector (pMD2.G), was cotransfected into human embryonic kidney 293T cells using polyethylenimine. Supernatants containing virus particles were collected at 48 h post-transfection and filtered through 0.45 μm filters to remove cell debris. The viruses were used to infect target cells grown in medium supplemented with 8 μg/ml polybrene. Infected cells were then obtained in the presence of 1 μg/ml puromycin. The shRNA targeting sequences used were shUBR1-1#, GCGTTGAGTCTTCGATTAAAT; shUBR1-2#, CCAAGAGACTAATCAGATGTT; shUBR2-1#, CCTCCTTACCTTGATGACTAT; shUBR2-2#, GCCGGAATGTGGAGAAGAAAT; shUBR4-1#, CCACATACATTGTTCGGGAAA; shUBR4-2#, CCACCATCAAAGACTTACATT; shUBR5-1#, TTGGAACAGGCTACTATTAAA; and shUBR5-2#, GCTGTAGATTTCAACTTAGAT.

### The ubiquitination assay

Ubiquitination of BCR–ABL was detected using denatured immunoprecipitation. Cells were lysed in SDS-denaturing buffer (50 mM Tris–HCl [pH 7.4], 150 mM NaCl, 1% NP-40, 1% SDS, and 0.5% sodium deoxycholate) and boiled for 10 min. Cell lysates were then diluted 10-fold in native lysis buffer (50 mM Tris–HCl [pH 7.4], 150 mM NaCl, 1% NP-40, and 0.5% sodium deoxycholate). After centrifugation at 15,000 rpm for 10 min, the supernatants were immunoprecipitated using glutathione-Sepharose 4B beads (GE Healthcare) and incubated for 2 h at 4 °C. Beads were then washed five times using the native lysis buffer. The immunoprecipitates were boiled with 2× SDS-PAGE loading buffer, separated on SDS-PAGE gels, followed by immunoblotting with various antibodies indicated.

### Cell viability assay

K562 cells (5 × 10^3^ cells per well) were grown in 96-well plates with 100 μl medium. After the treatment with the PROTACs at various concentrations (0, 0.01, 0.1, 0.25, 0.5, 0.75, 1, and 2.5 nM) for 48 h, CCK-8 solution was added to each well and incubated for 2 h at 37 °C, and the number of viable cells was determined by measuring the absorbance of the converted dye at a 450 nm wavelength, according to the manufacturer’s instructions (Yeasen). Each experiment was performed at least three times independently.

### Mouse xenograft models

K562 cells (1 × 10^7^ cells/200 μl) were subcutaneously injected into the right flank of 4-week-old female nude mice. Six mice were divided randomly and equally into two groups: vehicle, Arg-PEG1-Dasa, or Dasa (three mice per group), when the tumors reached a palpable volume of about 50 to 80 mm^3^ at day 7. The drugs were subsequently administered by i.p. injection every other day with either vehicle, Arg-PEG1-Dasa, or Dasa (10 mg/kg) for total 10 doses. The growth of tumors in mice was measured with a Vernier caliper. The tumor volume was determined using the formula volume = 0.5 × (length) × (width)^2^. Mice-bearing tumors were sacrificed at day 27 post-transplantation and then the tumors were excised and imaged. All animal experiments were approved by the Animal Care Committee of Southern University of Science and Technology.

### Immunohistochemical staining

Immunohistochemical staining was carried out to assay BCR–ABL on the K562 xenograft tumor sections. Specifically, the tissue sections were deparaffinized and rehydrated by heating the sample at 95 °C in Tris–EDTA buffer (pH 9.0) for 20 min. Endogenous peroxidase activity was blocked by peroxidase (ZSGB-BIO). The sections were blocked with goat serum and then mixed with anti-c-Abl (CST; catalog no.: 2862, 1:50 dilution) at 4 °C overnight, followed by mixing secondary antibodies (Proteintech) for 1 h and developed with 3,3′-diaminobenzidine. Hematoxylin was used to counterstain the nuclei.

### Statistical analysis

The data represent the mean ± SD values of samples obtained from three independent experiments. We performed a two-tailed Student’s *t* test to determine statistically significant differences between two groups. In the statistical analysis, *p* < 0.05 is deemed as statistically significant.

## Data availability

All methods and data described in the article or supporting information are available on request by the first author or the corresponding author.

## Supporting information

This article contains supporting information.

## Conflict of interest

The authors declare that they have no conflicts of interest with the contents of this article.
