# Supplementary material for: Single amino acid–based PROTACs trigger degradation of the oncogenic kinase BCR–ABL in chronic myeloid leukemia (CML)
Source: J Biol Chem. 2023 Jun 29;299(8):104994. doi: 10.1016/j.jbc.2023.104994 (PMC10388202; doi:10.1016/j.jbc.2023.104994)
Supplement: Supporting information [file mmc1.pdf]

Single amino acid-based PROTACs trigger degradation of the oncogenic kinase BCR-ABL in chronic myeloid leukemia (CML)

Jianchao Zhang[1,4], Caibing Ma[2,4], Yongjun Yu[1], Chaowei Liu[1], Lijing Fang[2*], Hai Rao[1,3*]

[1] Department of Biochemistry, School of Medicine, Southern University of Science and Technology, Shenzhen 518055, China

[2] Institute of Biomedicine and Biotechnology, Shenzhen Institute of Advanced Technology, Chinese Academy of Sciences, Shenzhen 518055, Guangdong, China.

[3] Key University Laboratory of Metabolism and Health of Guangdong, Southern University of Science and Technology, Shenzhen 518055, China

[4] These authors contributed equally to this work.

PROTACs synthesis and mass spectrometry identification

Synthesis and quality analysis of various PROTAC molecules are described here. We first synthesized Fmoc-PEGn-Dasatinib with varying numbers of PEG, which were then combined with different amino acids (e.g., Arg, Lys, Leu, Phe). The quality of these PROTAC compounds were subsequently analyzed by HMRS and HPLC.

1 General procedure A: the synthesis of Fmoc-PEG$_n$-Dasa

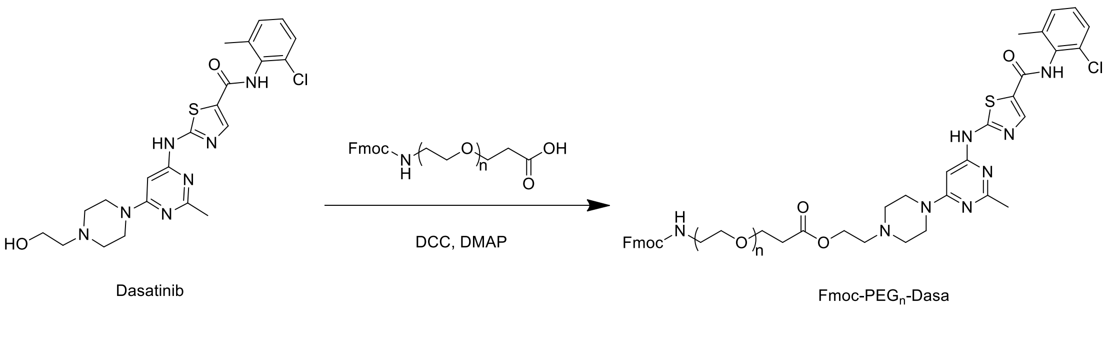

Dasatinib (1.0 eq) and Fmoc-PEGn-$(CH_2)_2$-COOH (1.5 eq) were dissolved in anhydrous DMF, a solution of DCC (1.5 eq) and DMAP (0.2 eq) in DCM was added. The mixture was stirred at room temperature for 4 hours and monitored by HPLC, and

then the solvent was removed under reduced pressure. The residue was purified by RP-HPLC to generate Fmoc-PEGn-Dasa after freeze-drying.

1.1 Synthesis of Fmoc-PEG1-Dasa

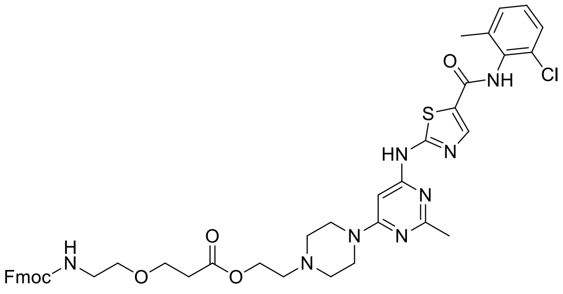

Fmoc-PEG1-Dasa

According to General procedure A, Fmoc-PEG1-Dasa was generated as a white powder.

1.2 Synthesis of Fmoc-PEG2-Dasa

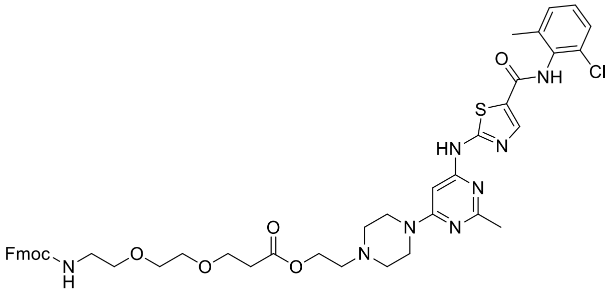

Fmoc-PEG2-Dasa

According to General procedure A, Fmoc-PEG2-Dasa was generated as a white powder.

1.3 Synthesis of Fmoc-PEG3-Dasa

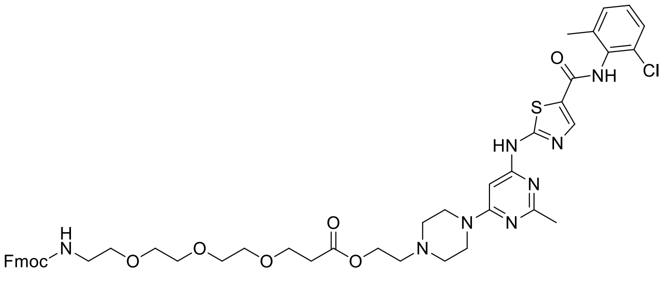

Fmoc-PEG3-Dasa

According to General procedure A, Fmoc-PEG3-Dasa was generated as a white powder.

1.4 Synthesis of Fmoc-PEG4-Dasa

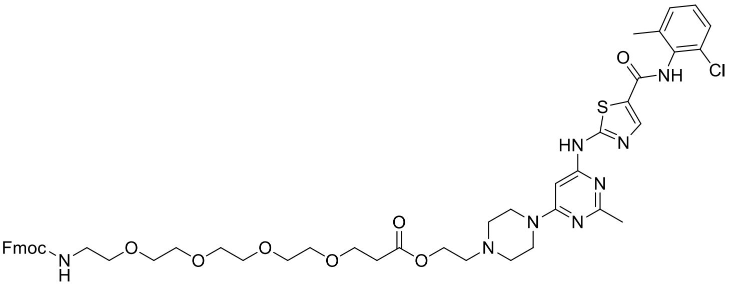

Fmoc-PEG4-Dasa

According to General procedure A, Fmoc-PEG4-Dasa was generated as a white powder.

2 General procedure B: the synthesis of Arg-PEG$_n$-Dasa

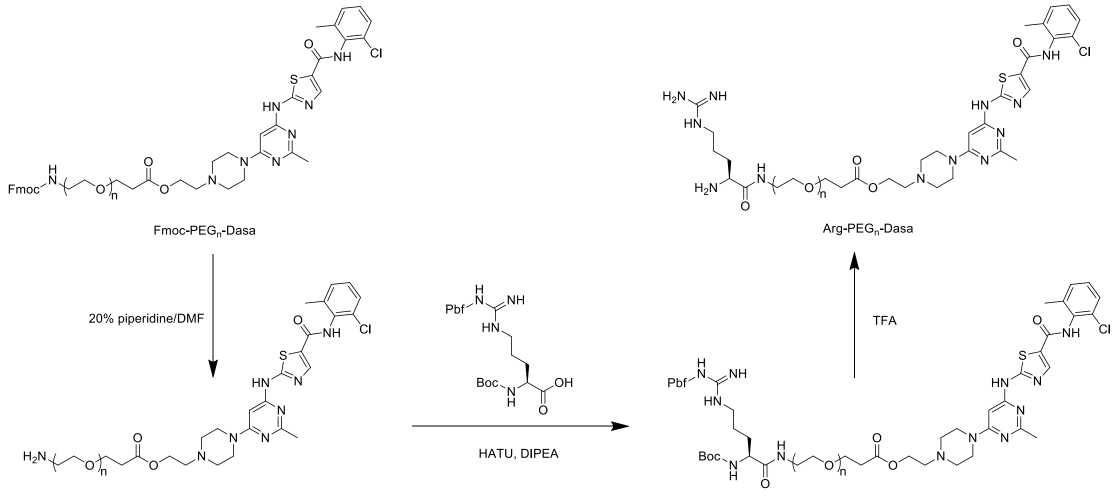

Fmoc-PEG$_n$-Dasa (1.0 eq) was dissolved in a solution of 20% piperidine in DMF, the mixture was stirred at room temperature for 1 hour and monitored by HPLC. Then the solvent was removed under reduced pressure. The residue was dissolved in DMF, Boc-Arg(Pbf)-OH (1.2 eq), DIPEA (3.0 eq), HATU (1.2 eq) were added successively. The mixture was stirred at room temperature for 2 hours and purified by RP-HPLC to generate Boc-Arg(Pbf)-PEG$_n$-Dasa. The protective groups were removed in a solution of 95% TFA in DCM for 0.5 hour, the solvent was removed under reduced pressure and the residue was purified by RP-HPLC to generate Arg-PEG$_n$-Dasa after freeze-drying.

2.1 Synthesis of Arg-PEG1-Dasa

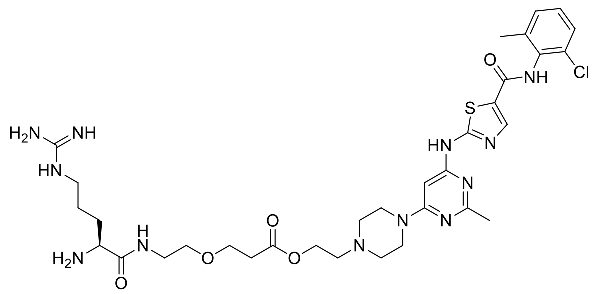

Arg-PEG1-Dasa

According to General procedure B, Arg-PEG1-Dasa was generated as a white powder (5.54 mg, 73%). HRMS (ESI) m/z: calcd. for $C_{33}H_{48}ClN_{12}O_5S$ $[M + H]^+$ 759.3274, found 759.32794.

2.2 Synthesis of Arg-PEG2-Dasa

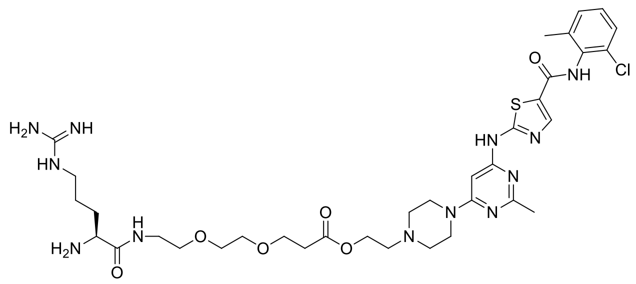

Arg-PEG2-Dasa

According to General procedure B, Arg-PEG2-Dasa was generated as a white powder (8.06 mg, 53%). HRMS (ESI) m/z: calculated for $C_{35}H_{51}ClN_{12}O_6S$ $[M + H]^+$ 803.3537, found 803.35297.

2.3 Synthesis of Arg-PEG3-Dasa

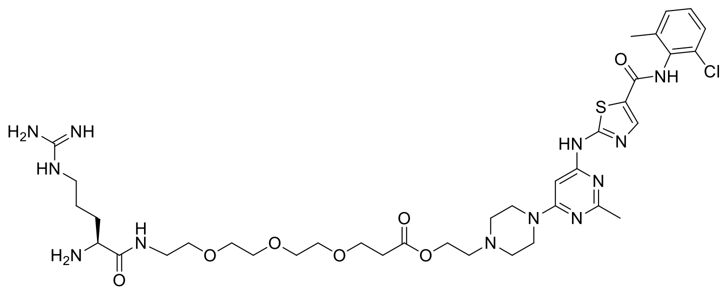

Arg-PEG3-Dasa

According to General procedure B, Arg-PEG3-Dasa was generated as a white powder (4.91 mg, 53%). HRMS (ESI) m/z: calculated for $C_{37}H_{55}ClN_{12}O_7S$ $[M + H]^+$ 847.3799, found 847.37909.

2.4 Synthesis of Arg-PEG4-Dasa

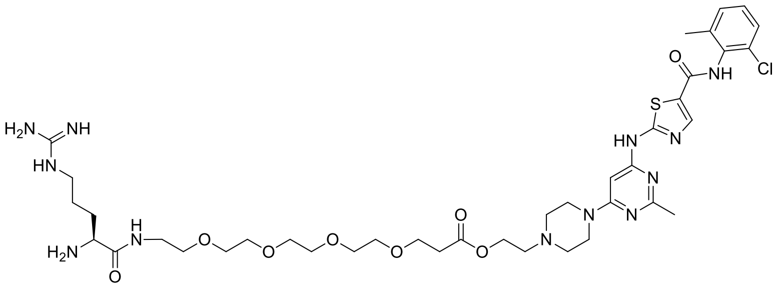

Arg-PEG4-Dasa

According to General procedure B, Arg-PEG4-Dasa was generated as a white powder (7.50 mg, 44%). HRMS (ESI) m/z: calculated for $C_{39}H_{59}ClN_{12}O_8S$ $[M + H]^+$ 891.4061, found 891.40521.

2.5 Synthesis of NH2-PEG1-Dasa

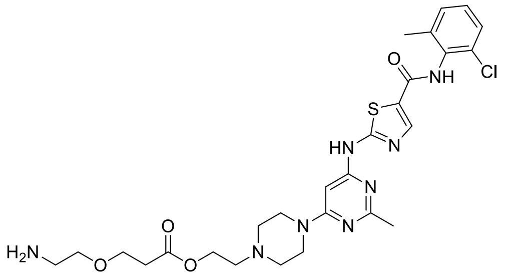

NH2-PEG1-Dasa

According to General procedure B, NH2-PEG1-Dasa was generated as a white powder (5.78 mg, 96%). HRMS (ESI) m/z: calculated for $C_{27}H_{35}ClN_8O_4S$ $[M + H]^+$ 603.2263, found 603.22597.

2.6 Synthesis of Lys-PEG1-Dasa

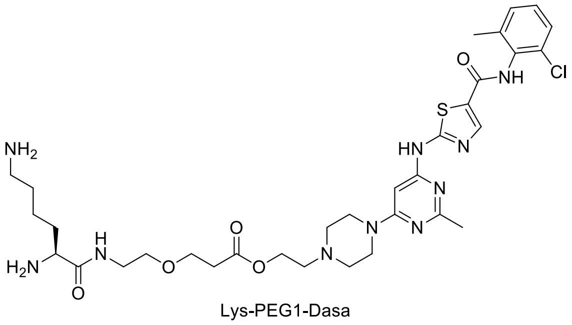

Lys-PEG1-Dasa

Similar with General procedure B, Fmoc-PEG1-Dasa (19.2 mg, 0.023 mmol, 1.0 eq) was dissolved in a solution of 20% piperidine in DMF (0.5 mL) and stirred at room temperature for 1 hour, then the solvent was removed. The residue was dissolved in

DMF (1 mL), Fmoc-Lys(Boc)-OH (13.1 mg, 0.028 mmol, 1.2 eq), DIPEA (11.4 μL, 0.069 mmol, 3.0 eq), HATU (10.6 mg, 0.028 mmol, 1.2 eq) were added successively. The mixture was stirred at room temperature for 2 hours and purified by RP-HPLC to generate Fmoc-Lys(Boc)-PEG1-Dasa after freeze-drying. The product was dissolved in a solution of 50% diethylamine in DCM (1 mL) and stirred at room temperature for 1 hour, then the solvent was removed under reduced pressure. The residue was dissolved in a solution of 50% TFA in DCM (1 mL) and stirred at room temperature for 1 hour. The solvent was removed under reduced pressure and the residue was purified by RP-HPLC to generate Lys-PEG1-Dasa as a white powder after freeze-drying (6.04 mg, 36%). HRMS (ESI) m/z: calculated for $C_{33}H_{47}ClN_{10}O_5S$ $[M + H]^+$ 731.3213, found 731.32147.

2.7 Synthesis of Leu-PEG1-Dasa

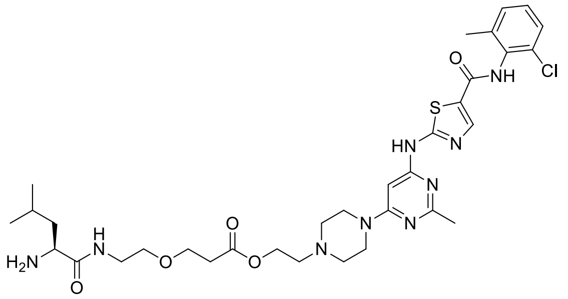

Leu-PEG1-Dasa

Similar with General procedure B, Fmoc-PEG1-Dasa (8.2 mg, 0.01 mmol, 1.0 eq) was dissolved in a solution of 20% piperidine in DMF (0.5 mL), the mixture was stirred at room temperature for 1 hour and monitored by HPLC. Then the solvent was removed under reduced pressure. The residue was dissolved in DMF (0.5 mL), Fmoc-Leu-OH (4.3 mg, 0.012 mmol, 1.2 eq), DIPEA (5.0 μL, 0.03 mmol, 3.0 eq), HATU (4.6 mg, 0.012 mmol, 1.2 eq) were added successively. The mixture was stirred at room temperature for 2 hours and purified by RP-HPLC to generate Fmoc-Leu-PEG1-Dasa as a white powder after freeze-drying. Fmoc-Leu-PEG1-Dasa was dissolved in a solution of 50% diethylamine in DCM (1 mL) and stirred at room temperature for 1 hour, then the solvent was removed by rotary evaporator under reduced pressure. The residue was purified by RP-HPLC to generate Leu-PEG1-Dasa as a white powder after

freeze-drying (5.66 mg, 79%). HRMS (ESI) m/z: calculated for $C_{33}H_{46}ClN_9O_5S$ [M + H]$^+$ 716.3106, found 716.31055.

2.8 Synthesis of Phe-PEG1-Dasa

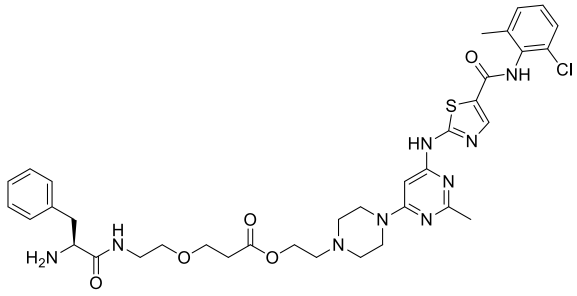

Phe-PEG1-Dasa

Similar with General procedure B, Fmoc-PEG1-Dasa (16.4 mg, 0.02 mmol, 1.0 eq) was dissolved in a solution of 20% piperidine in DMF (0.5 mL), the mixture was stirred at room temperature for 1 hour and monitored by HPLC. Then the solvent was removed under reduced pressure. The residue was dissolved in DMF (1 mL), Fmoc-Phe-OH (9.3 mg, 0.024 mmol, 1.2 eq), DIPEA (9.9 μL, 0.06 mmol, 3.0 eq), HATU (9.1 mg, 0.024 mmol, 1.2 eq) were added successively. The mixture was stirred at room temperature for 2 hours and purified by RP-HPLC to generate Fmoc-Phe-PEG1-Dasa as a white powder after freeze-drying. Fmoc-Phe-PEG1-Dasa was dissolved in a solution of 50% diethylamine in DCM (1 mL) and stirred at room temperature for 1 hour, then the solvent was removed under reduced pressure. The residue was purified by RP-HPLC to generate Phe-PEG1-Dasa as a white powder after freeze-drying (9.52 mg, 64%). HRMS (ESI) m/z: calculated for $C_{36}H_{45}ClN_9O_5S$ [M + H]$^+$ 750.2947, found 750.29340.

3 HRMS Spectra

3.1 HRMS spectrum of Arg-PEG1-Dasa

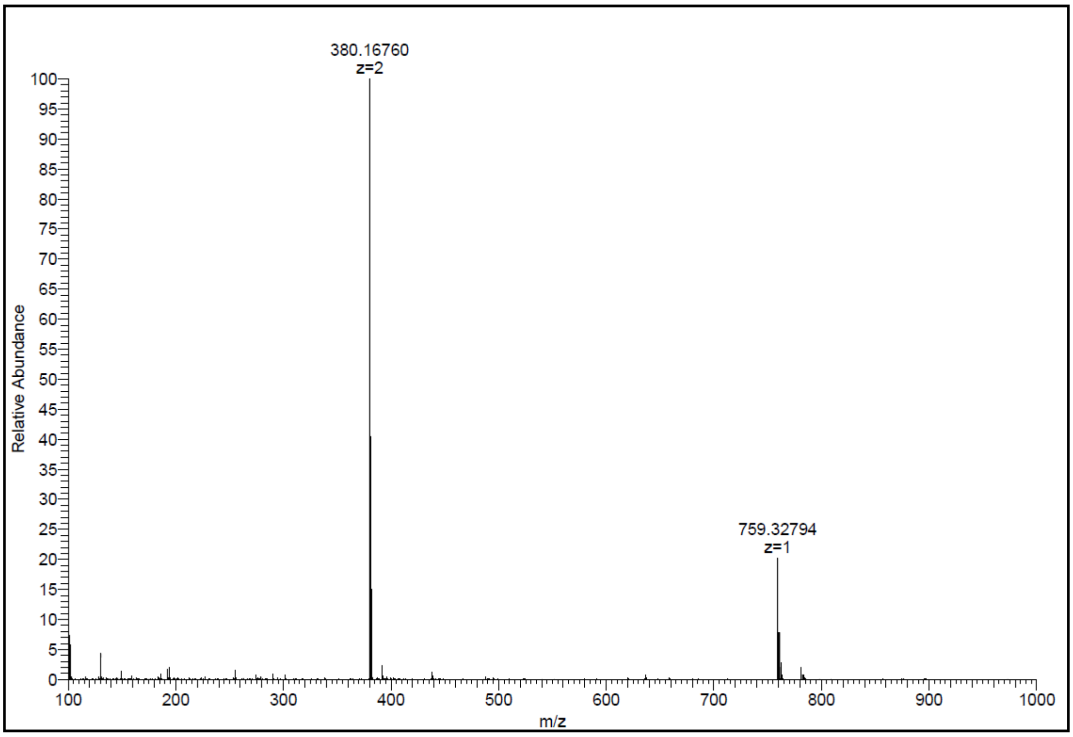

3.2 HRMS spectrum of Arg-PEG2-Dasa

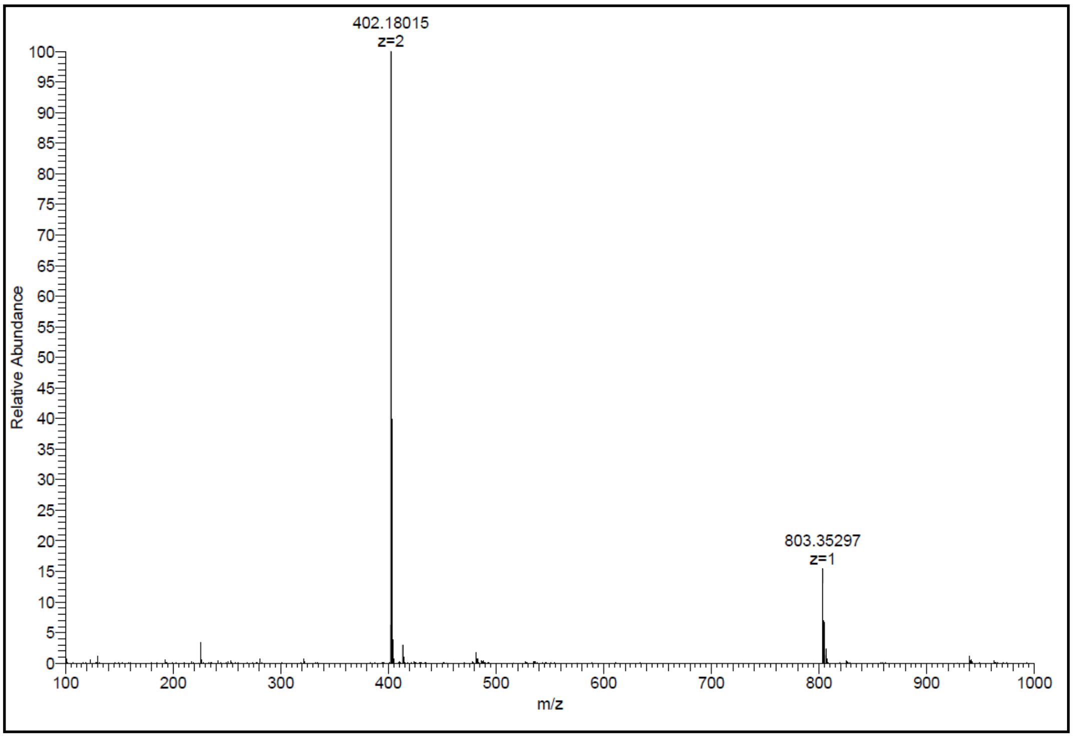

3.3 HRMS spectrum of Arg-PEG3-Dasa

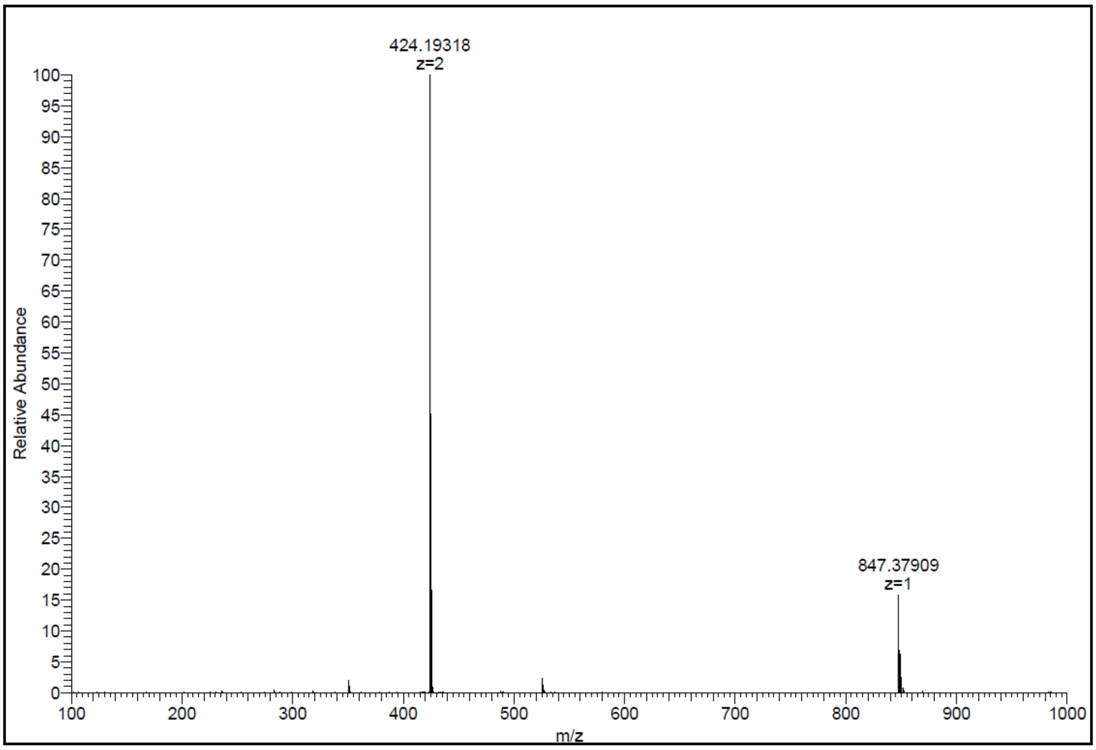

3.4 HRMS spectrum of Arg-PEG4-Dasa

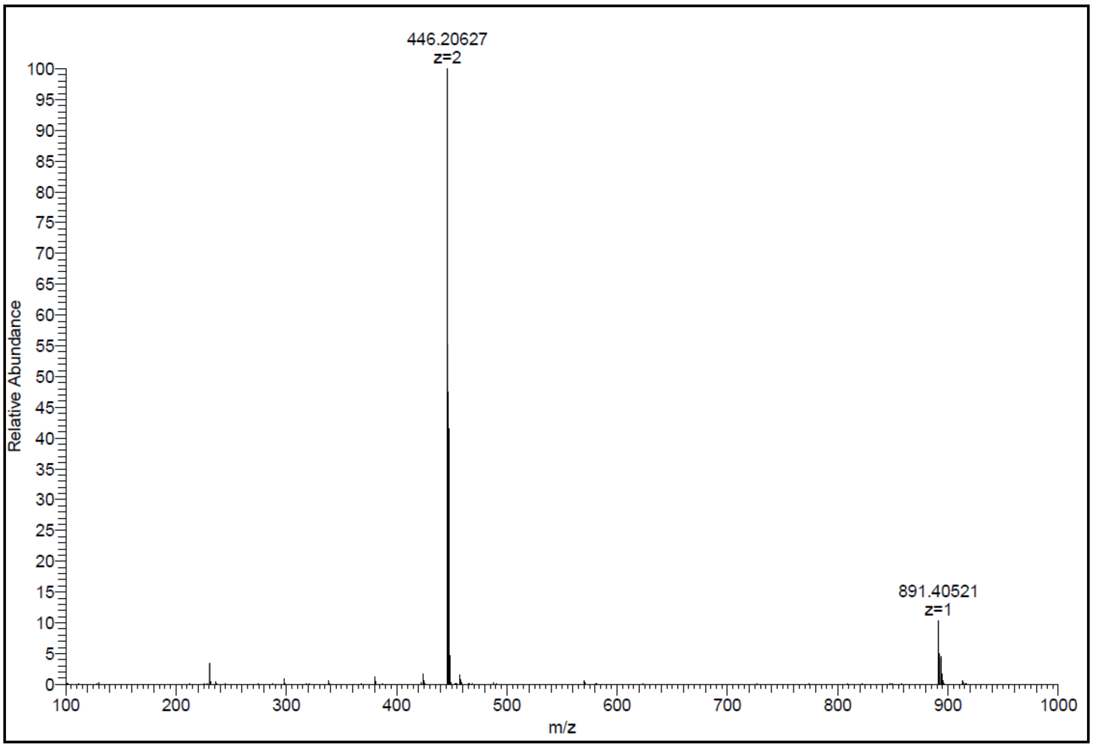

3.5 HRMS spectrum of NH2-PEG1-Dasa

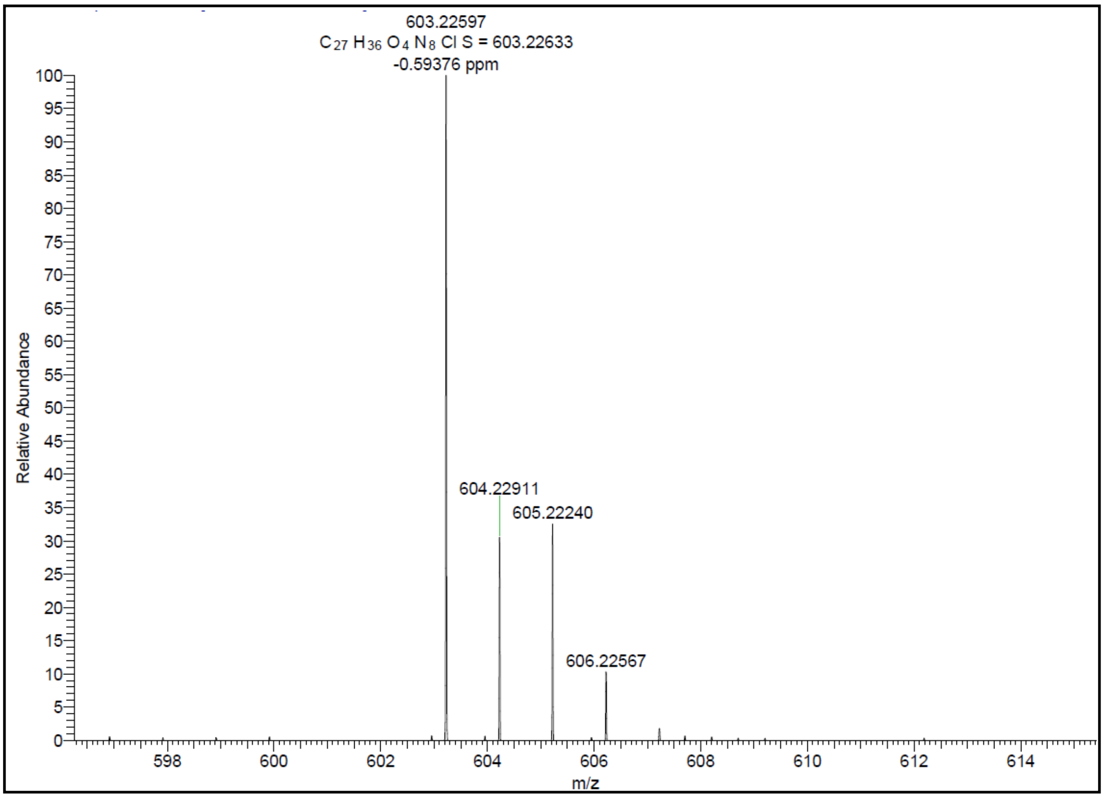

3.6 HRMS spectrum of Lys-PEG1-Dasa

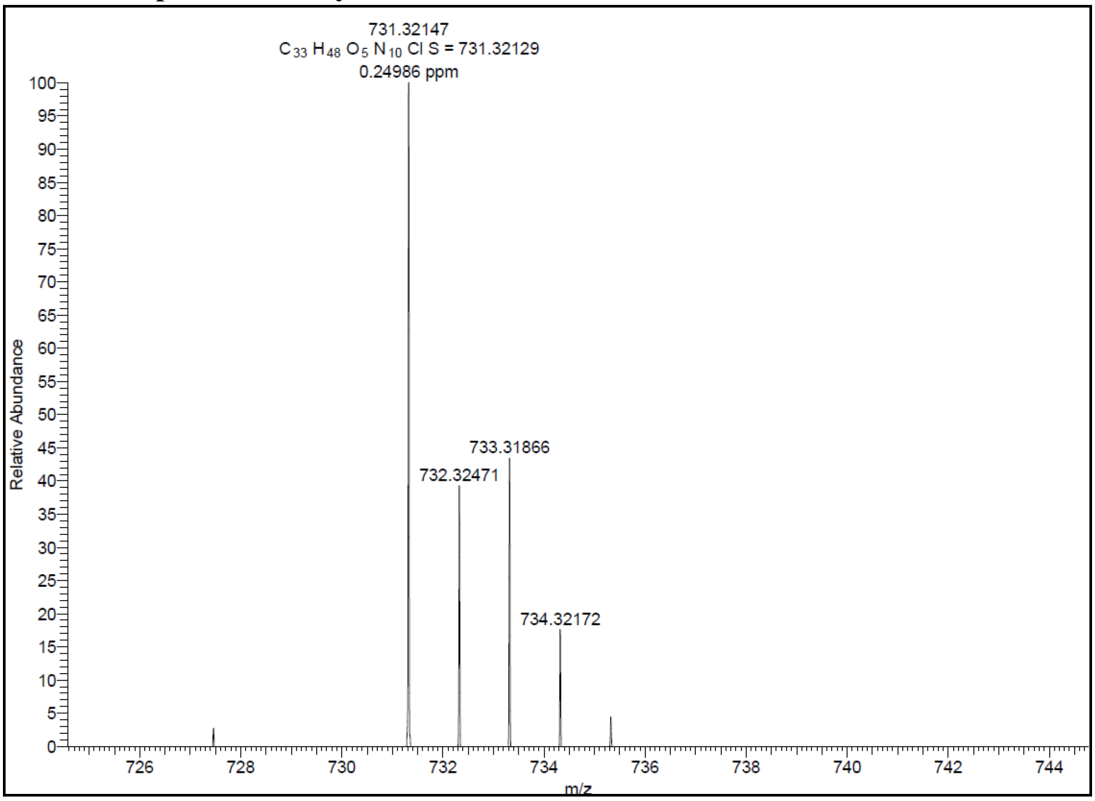

3.7 HRMS spectrum of Leu-PEG1-Dasa

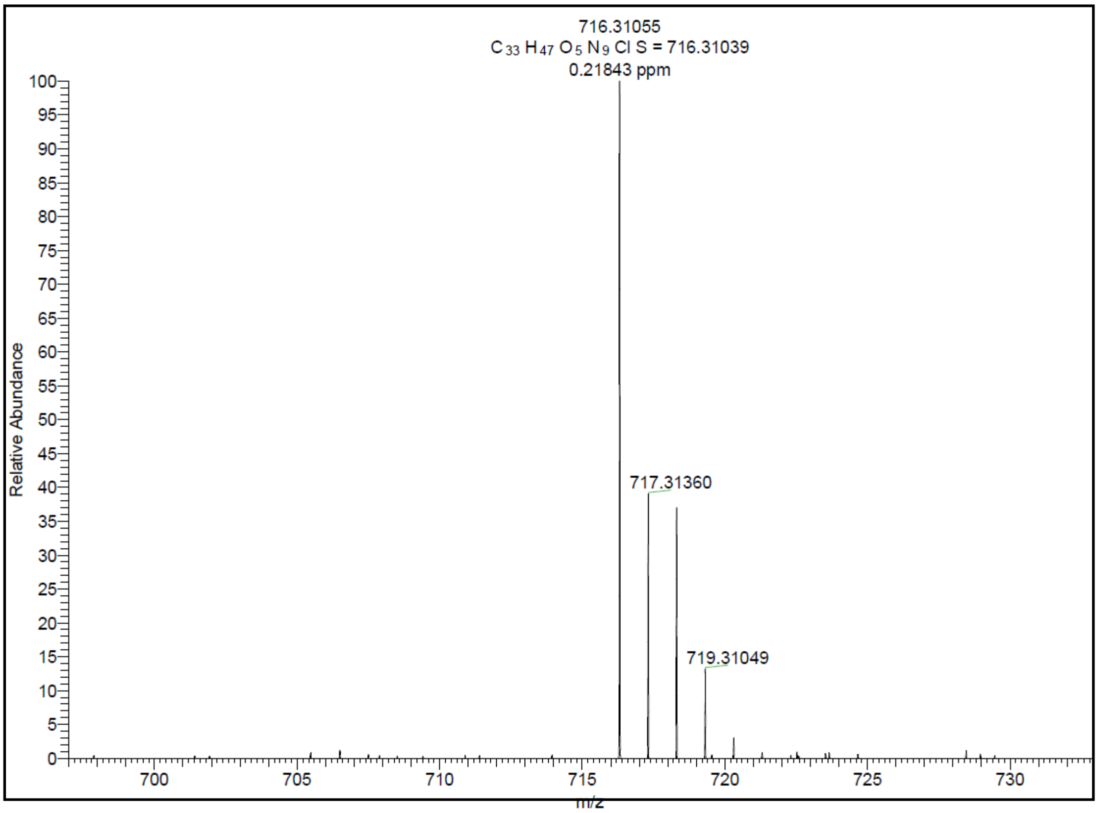

3.8 HRMS spectrum of Phe-PEG1-Dasa

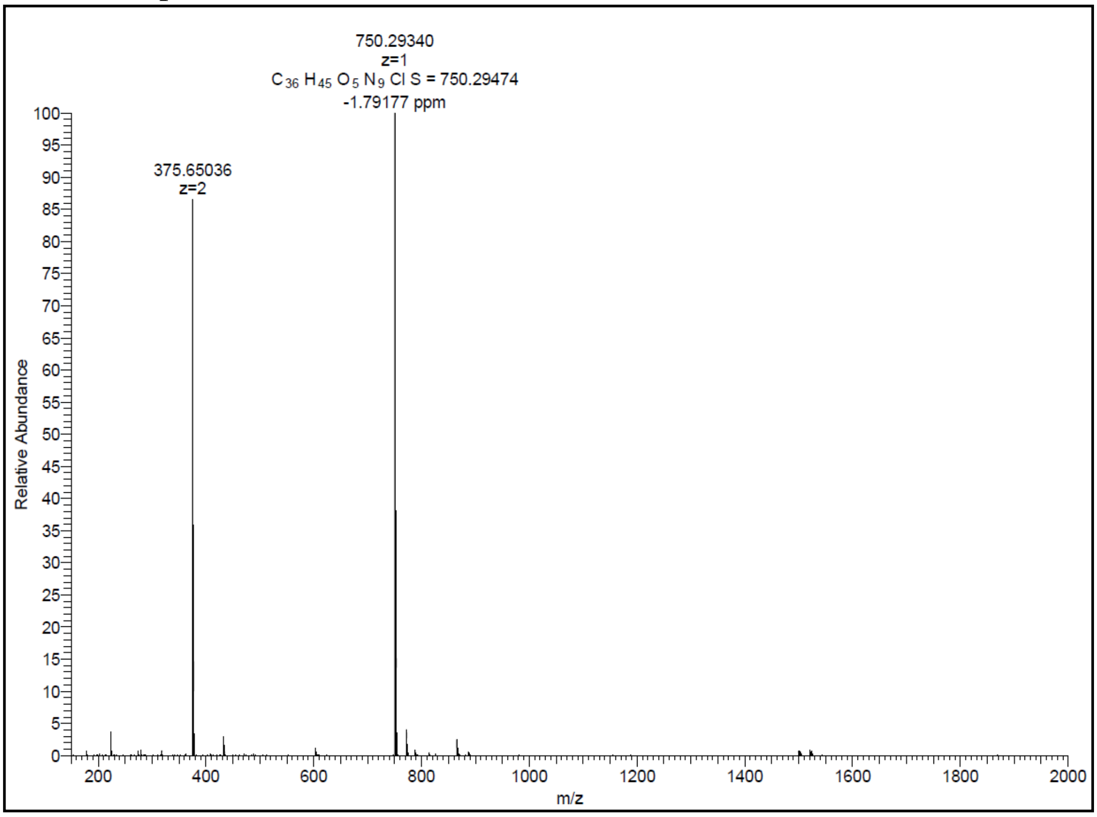

4 HPLC Spectra

Sig=220 nm Result

4.1 HPLC analysis of Arg-PEG1-Dasa

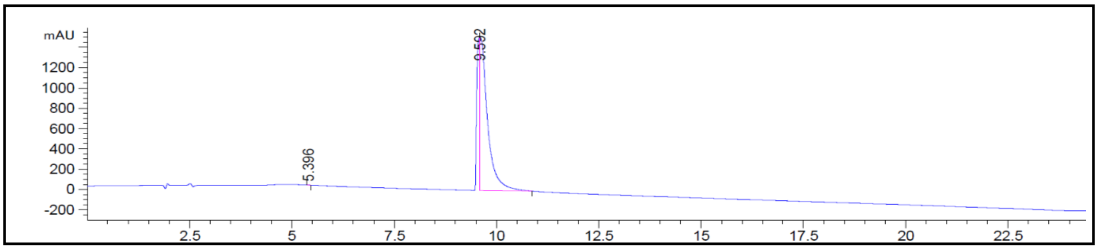

Peak #	Retention Time (min)	Height (mAU)	Area (mAU*s)	Area%
1	5.396	1.94553	7.33711	0.0359
2	9.592	1514.29846	20455.30	99.9641
Totals		1516.24399	20462.63711	

4.2 HPLC analysis of Arg-PEG2-Dasa

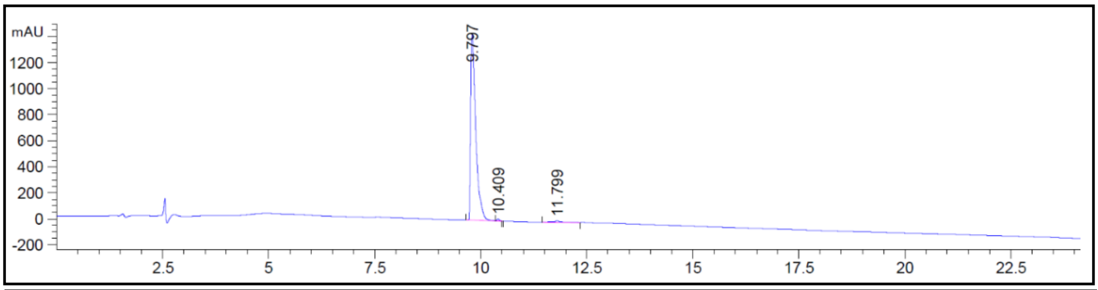

Peak #	Retention Time (min)	Height (mAU)	Area (mAU*s)	Area%
1	9.797	1432.74426	11832.30	97.4408
2	10.409	14.16072	52.78859	0.4347
3	11.799	13.76699	257.98291	2.1245
Totals		1460.67197	12143.0715	

4.3 HPLC analysis of Arg-PEG3-Dasa

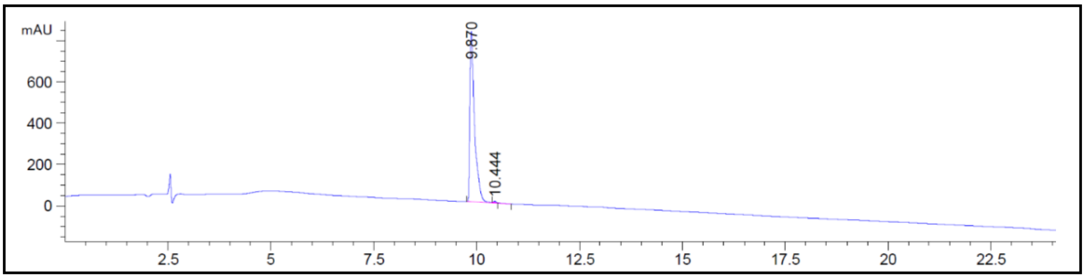

Peak #	Retention Time (min)	Height (mAU)	Area (mAU*s)	Area%
1	9.870	824.55542	6624.65381	99.5980
2	10.444	7.09019	26.73623	0.4020
Totals		831.64561	6651.39004	

4.4 HPLC analysis of Arg-PEG4-Dasa

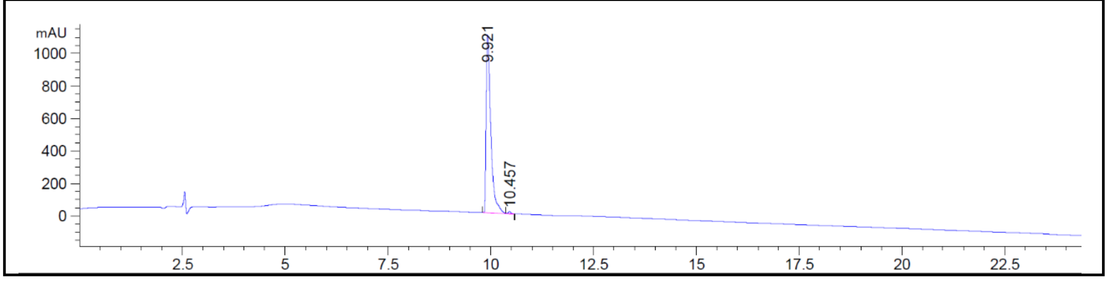

Peak #	Retention Time (min)	Height (mAU)	Area (mAU*s)	Area%
1	9.921	1097.66406	8661.20996	99.3844
2	10.457	13.39128	53.64834	0.6156
Totals		1111.05534	8714.8583	

4.5 PLC analysis of NH_2-PEG1-Dasa

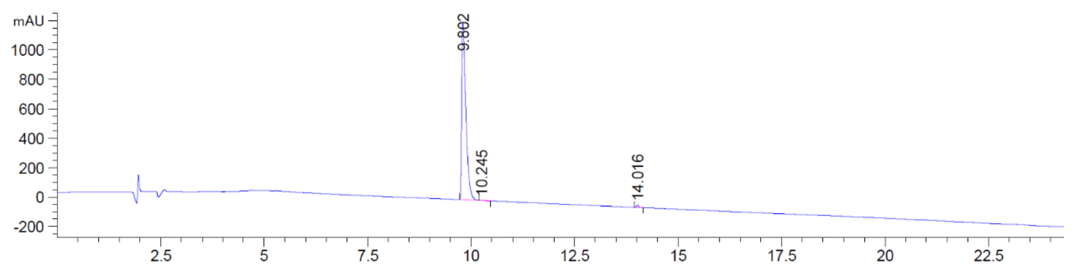

Peak #	Retention Time (min)	Height (mAU)	Area (mAU*s)	Area%
1	9.802	1203.51221	8242.2002	99.0781
2	10.245	1.68651	9.37344	0.1127
3	14.016	16.47169	67.32124	0.8093
Totals		1221.67041	8318.89488	

4.6 HPLC analysis of Lys-PEG1-Dasa

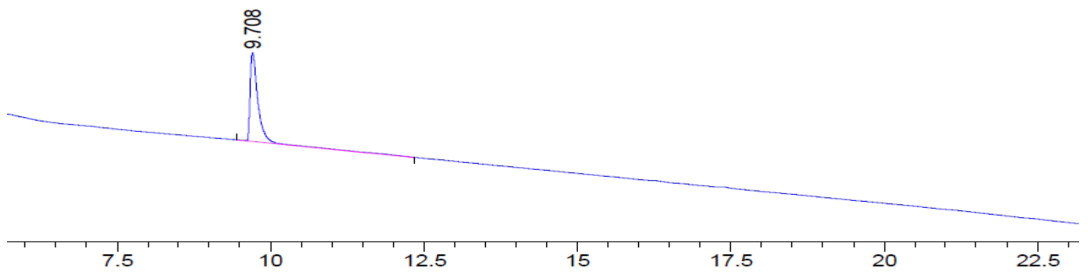

Peak #	Retention Time (min)	Height (mAU)	Area (mAU*s)	Area%
1	9.708	194.34427	1746.69214	100.0000
Totals		194.34427	1746.69214	

4.7 HPLC analysis of Leu-PEG1-Dasa

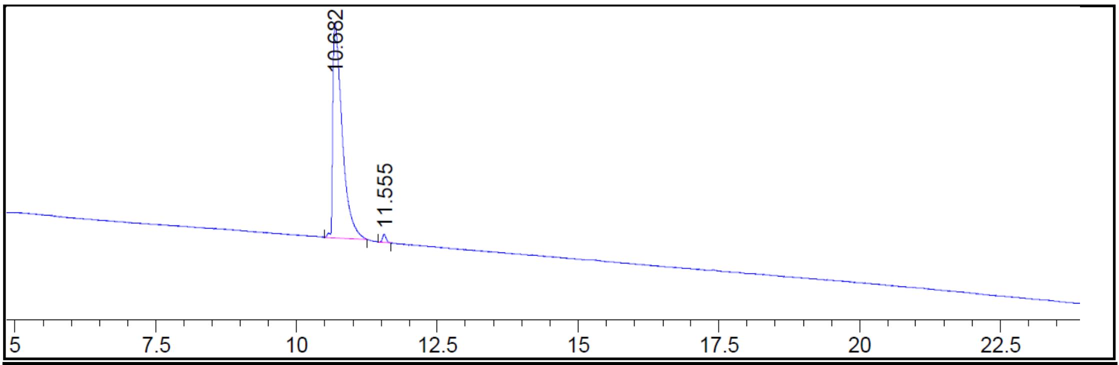

Peak #	Retention Time (min)	Height (mAU)	Area (mAU*s)	Area%
1	10.682	514.82782	5954.68164	98.5478
2	11.555	19.53011	87.74986	1.4522
Totals		534.35793	6042.4315	

4.8 HPLC analysis of Phe-PEG1-Dasa

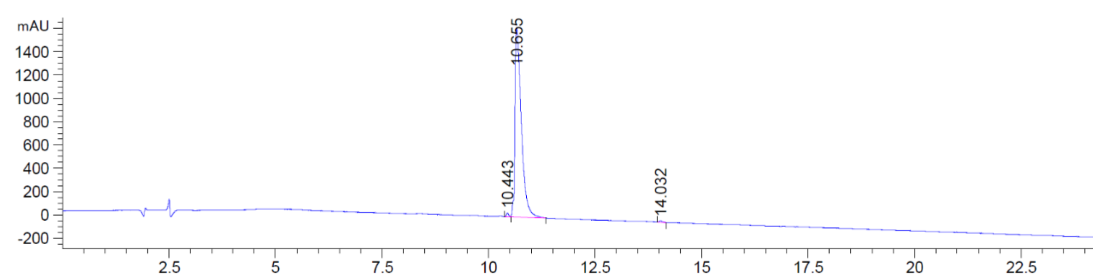

Peak #	Retention Time (min)	Height (mAU)	Area (mAU*s)	Area%
1	10.443	32.98214	120.54449	0.7096
2	10.655	1620.12622	16799.50	98.8984
3	14.032	14.77383	66.58022	0.392
Totals		1667.88219	16986.62471	

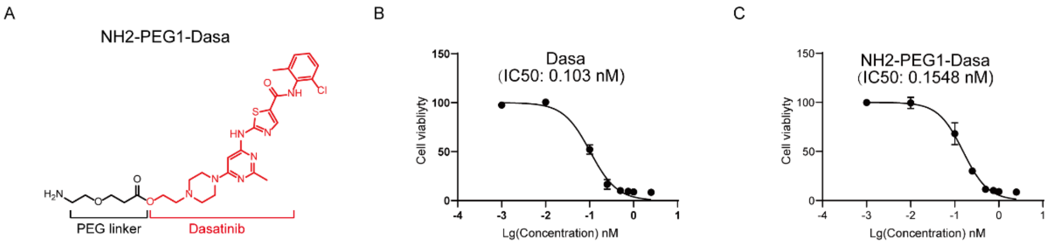

Figure S1. Dasatinib (Dasa) or NH2-PEG1-Dasa-mediated inhibition of cell proliferation in K562 cells

(A) Schematic of NH2-PEG1-Dasa. (B-C) K562 cells viability was examined using CCK-8 assays after the treatment with Dasa (B) or NH2-PEG1-Dasa (C) for 48 hours at various concentrations (0, 0.01, 0.1, 0.25, 0.5, 0.75, 1, 2.5 nM).
